# Translocation across a human enteroid monolayer by zoonotic *Streptococcus suis* correlates with the presence of Gb3-positive cells

**DOI:** 10.1016/j.isci.2024.109178

**Published:** 2024-02-12

**Authors:** Thomas J. Roodsant, Kees C.H. van der Ark, Constance Schultsz

**Affiliations:** 1 Amsterdam UMC, Location University of Amsterdam, Department of Global Health, Amsterdam Institute for Global Health and Development, Meibergdreef 9, Amsterdam, the Netherlands; 2 Amsterdam UMC, Location University of Amsterdam, Department of Medical Microbiology and Infection Prevention, Meibergdreef 9, Amsterdam, the Netherlands

**Keywords:** Medical microbiology, Microbiology, Molecular microbiology

## Abstract

*Streptococcus suis* is a zoonotic pathogen that can cause meningitis and septicaemia. The consumption of undercooked pig products is an important risk factor for zoonotic infections, suggesting an oral route of infection. In a human enteroid model, we show that the zoonotic CC1 genotype has a 40% higher translocation frequency than the non-zoonotic CC16 genotype. Translocation occurred without increasing the permeability or disrupting the adherens junctions and tight junctions of the epithelial monolayer. The translocation of zoonotic *S. suis* was correlated with the presence of Gb3-positive cells, a human glycolipid receptor found on Paneth cells and targeted by multiple enteric pathogens. The virulence factors Streptococcal adhesin Protein and suilysin, known to interact with Gb3, were not essential for translocation in our epithelial model. Thus, the ability to translocate across an enteroid monolayer correlates with *S. suis* core genome composition and the presence of Gb3-positive cells in the intestinal epithelium.

## Highlights

• *S. suis* SS2CC1 translocates more across enteroid monolayers than SS2CC20 and SS9CC16
• *S. suis* translocation occurs without the disruption of the enteroid monolayer
• Gb3-positive cells are present within human enteroid monolayers
• Zoonotic *S. suis* translocation correlates with Gb3-positive cell presence

## Graphical abstract

**Figure:**
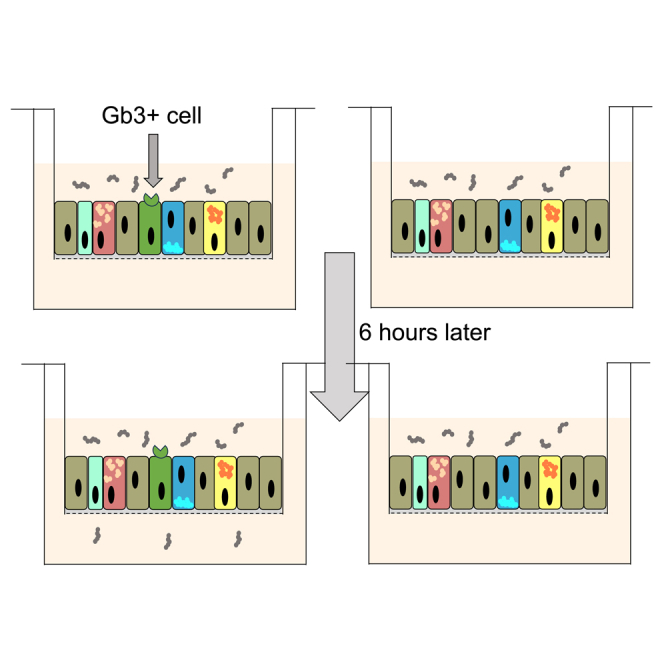

## Introduction

*Streptococcus suis* is an opportunistic pathogen in pigs and a zoonotic pathogen that causes meningitis, streptococcal toxic-shock such as syndrome and septicaemia.^1^^,^^2^^,^^3^^,^^4^ *S. suis* strains are typed into serotypes based on the antigenic structure of their capsular polysaccharide and into clonal complexes based on their genotypes. Zoonotic infections are almost exclusively caused by *S. suis* serotype 2 (SS2) strains from predominantly clonal complex 1 (CC1), although human infections with strains belonging to other CC such as CC20, CC25, CC28, and CC104 have also been reported.^3^ In the Netherlands, the zoonotic SS2CC20 genotype has emerged from the non-zoonotic but virulent pig SS9CC16 genotype.^5^^,^^6^ In Thailand and Vietnam, where SS2CC1 is the dominant cause of zoonotic *S. suis* infections, the consumption of raw or undercooked pig products is an important risk factor for human *S. suis* infection^2^^,^^4^^,^^7^^,^^8^ and SS2 were detected by PCR in the throat and rectal swabs of Vietnamese patients with *S. suis* infection but not in healthy controls.^8^ These data indicate that *S. suis* should be considered a foodborne pathogen and suggest that the gastrointestinal tract is a potential entry site for *S. suis*, resulting in systemic infection in the human host.

In a pig oral challenge model, pigs were fed with a gastric-acid resistant capsule containing zoonotic SS2CC1. Multiple pigs were successfully infected by *S. suis* and *S. suis* was detected in the jejunum (small intestine) and colon of an infected pig.^9^ Additionally, in a Caco-2 continuous cell culture model for the human intestinal epithelial, zoonotic *S. suis* SS2CC1 and SS2CC20 showed increased adhesion compared to non-zoonotic SS9CC16 strains. SS2CC1 showed a higher median percentage of translocation across a Caco-2 cell monolayers than SS9CC16, suggesting a possible correlation between the *S. suis* genotype and its potential as foodborne pathogen.^9^ The adhesion and translocation of SS2CC1 in the Caco-2 model were partly mediated by the surface anchored protein streptococcal adhesin protein (SadP), also known as factor H binding protein.^10^ Multiple *sadP* alleles have been described, which encode SadP proteins that vary in ligand specificity and anchoring to the bacterial cell wall.^10^^,^^11^ SadP of CC1 is cell wall anchored and can bind to the globoside lipids Gb3 (CD77) and Gb4, and the human blood group antigen P1.^10^^,^^11^ While SadP of CC16 and CC20 can also bind to Gb3, it cannot bind Gb4, and SadP of CC16 lacks a cell wall anchor.^10^^,^^11^ Gb3 is present *in vivo* on the surface of multiple human cell types, including cells of lung epithelium, kidney epithelium, and erythrocytes, as well as on Caco-2, Hep-2, HUVECs, and hCMEC/D3 cell lines cultured *in vitro*.^11^^,^^12^^,^^13^^,^^14^ In pigs, Gb3 was found at relative high levels in intestinal and lung tissue compared to other sampled tissues from different body sites.^15^ However, in the human gastrointestinal tract, Gb3 presence has been disputed, despite the fact that several human enteric pathogens use Gb3 as receptor for some of their virulence factors.^16^^,^^17^^,^^18^^,^^19^

Both *in vivo* pig models and *in vitro* Caco-2 cell line models have been used to study host*-S. suis* interactions,^20^^,^^21^ but both have their own limitations as models for human foodborne *S. suis* infections. Despite the great similarity between the pig and human intestines,^20^ there are differences in anatomy, cellular distribution, and receptor expression.^22^^,^^23^^,^^24^^,^^25^ Additionally, when studying zoonotic pathogens, it can be argued that the natural host is not the optimal model to study human infection. The cancer derived Caco-2 cell line is of human origin and, when differentiated, resembles small intestinal enterocytes, but lacks the multicellular complexity of the intestinal epithelium and shows deviating protein expression profiles compared to the human intestinal epithelium.^26^^,^^27^ Human intestinal explant models offer the multicellular complexity of the intestinal epithelium and the 3D architecture of the human intestine. Human intestinal explant models have been established and used to study host-pathogens interactions.^28^ In *S. suis* research, *ex vivo* pig models such as precision-cut long slices have been used to study host*-S. suis* interactions.^29^ However, the difficulty of obtaining human tissue samples and the short lifespan of the explants are disadvantages of these models. The recent advances in organotypic cultures allow for studying bacterial pathogenesis in a multicellular model that mimics the cellular complexity of the human intestinal epithelium, such as enteroids.^24^^,^^30^ In contrast to explants, organoids including enteroids can be subcultered allowing for a multitude of experiments from a single tissue sample.^28^ Enteroids are primary cell derived organoids from the small intestine, consisting of epithelial cells. Enteroid monolayers grown on permeable supports have successfully been used to study host-pathogen interactions in the intestine.^19^^,^^24^^,^^31^^,^^32^^,^^33^^,^^34^^,^^35^^,^^36^ Recently, pluripotent stem cell derived intestinal organoids were shown to contain Gb3 and were used to study Shiga toxin toxicity.^19^ To further understand the role of the gastro-intestinal tract in the pathogenesis of zoonotic and potentially foodborne *S. suis* infection, we studied the adhesion, invasion, and translocation of zoonotic and non-zoonotic *S. suis* in a human enteroid model and assessed whether translocation was dependent on the presence of Gb3-positive cells.

## Results

### Zoonotic *S. suis* have a higher translocation frequency across proximal than distal derived enteroid monolayers

**Figure 1:**
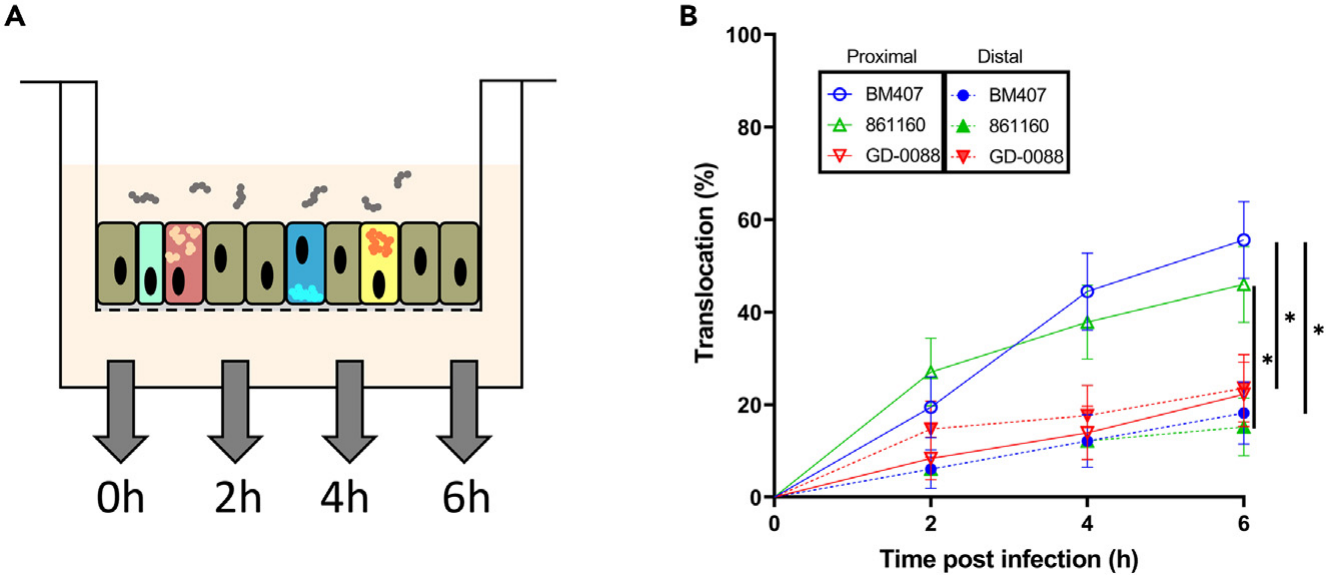
Zoonotic *S. suis* have a higher translocation frequency across proximal than distal derived enteroid monolayers (A) Graphical representation of the enteroid monolayer infection. (B) Enteroid monolayers were apically infected (MOI 50) with *S. suis* strain BM407 (SS2CC1), 861160 (SS2CC20) or GD-0088 (SS9CC16). Translocation events were recorded every 2 h by plating the basolateral medium and the frequency of translocation, expressed as the number of monolayers in which translocation occurred at 2, 4 and 6 h post infection, relative to the total number of monolayers infected, was plotted. Data from 5 donors were pooled, with at least 6 monolayers per donor. Open symbols indicate proximal enteroid monolayers; closed symbols distal enteroid monolayers. Statistical difference was determined using a log rank (Mantel-cox) test with a Bonferroni correction for multiple testing, error bars show SE and ∗p < 0.05.

The representative zoonotic SS2CC1 (BM407) and SS2CC20 (861160), and non-zoonotic SS9CC16 (GD-0088) *S. suis* strains, that were previously shown to translocate at different rates across a Caco-2 cell monolayer,^9^ were selected to study the translocation of *S. suis* across human enteroid monolayers derived from proximal (duodenum, jejunum) or distal (ileum) small intestine tissue. We previously observed differences in susceptibility to bacterial translocation between donors and along the intestinal tract^34^; therefore, we infected proximal and distal enteroid monolayers from 5 different donors in at least 3 independent experiments per donor (Figure 1A). As observed previously for *Listeria monocytogenes*, the translocation of the strains differed between donors (Figure S1). In addition, within each donor, the frequency of translocation showed variation between replicate monolayers, and between independent experiments (Figure S1), with replicate monolayers infected by the same strain within a single experiment showing the translocation of 10^3^-10^4^ CFUs or no translocation at all. To take into account this biological variation and data distribution within and across experiments, translocation was defined as present when detecting any count of CFUs in the plated basolateral medium, and the frequency of translocation was expressed as a proportion, i.e., the number of monolayers in which translocation occurred at 2, 4 and 6 h post infection (hpi), relative to the total number of monolayers infected. The zoonotic strains BM407 and 861160 had a higher translocation frequency (p = 0.013 and p = 0.046 respectively) across proximal (56% and 46% at 6 hpi respectively) than distal derived enteroid monolayers (18% and 15% at 6 hpi respectively), while the non-zoonotic strain GD-0088 showed a similar lower translocation frequency across both proximal and distal enteroid monolayers (22% and 24% at 6 hpi) (Figure 1B). The translocation frequency of zoonotic SS2CC1 strain BM407 (56% at 6 hpi) was significantly higher (p = 0.032) than the translocation frequency of non-zoonotic SS9CC16 strain GD-0088 across proximal enteroid monolayers (22% at 6 hpi) (Figure 1B). To limit biological variation, allowing for meaningful comparisons between different *S. suis* strains, only proximal enteroids from a single donor (Figure S1, donor #1) were used for the remaining experiments. The selected donor showed good growth and a moderate bacterial translocation compared to the organoids from other donors.

### The translocation frequency across proximal enteroid monolayers was higher for zoonotic *S. suis* than non-zoonotic *S. suis* without damaging barrier function

**Figure 2:**
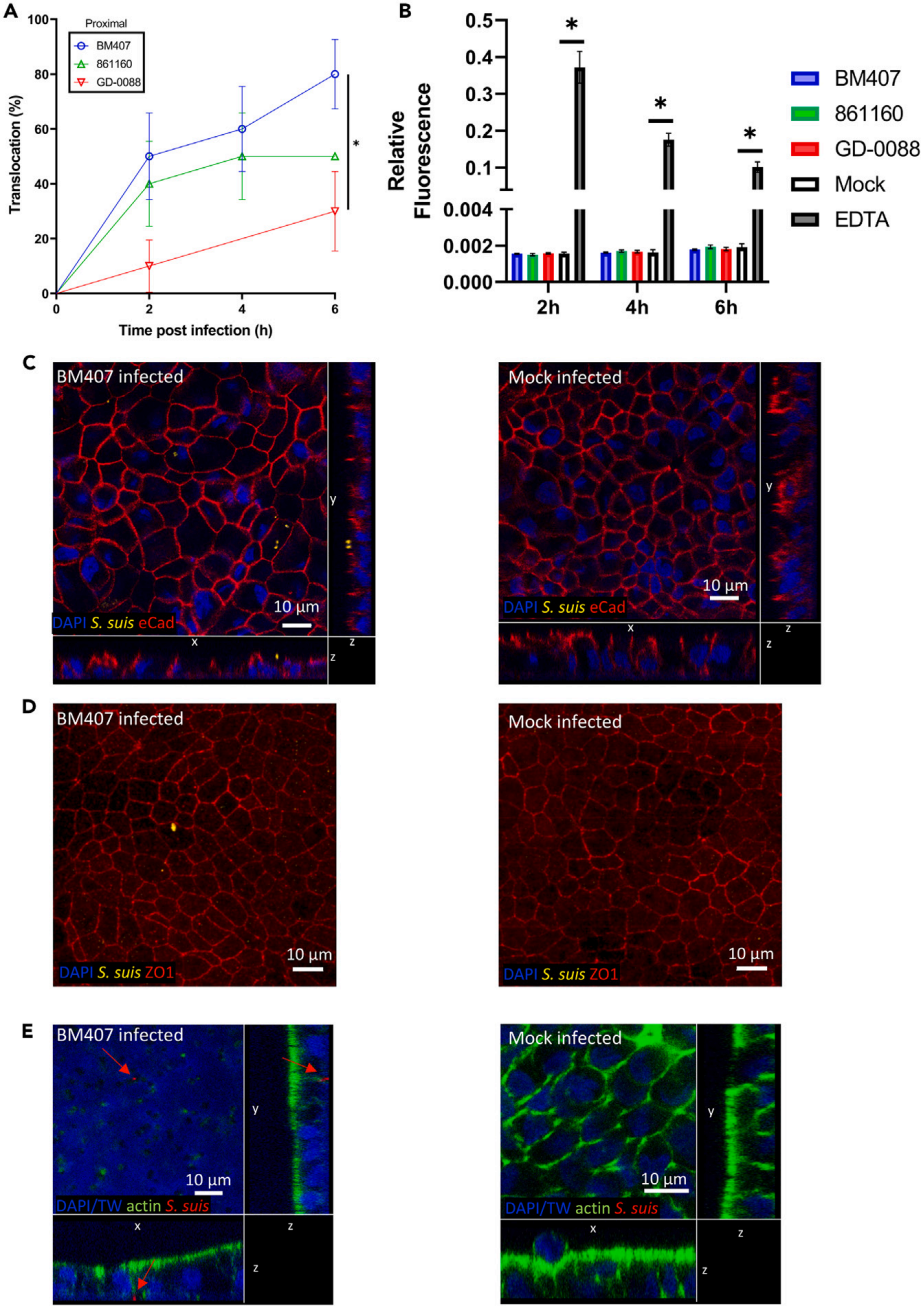
Zoonotic *S. suis* strain BM407 has a higher translocation frequency across proximal enteroid monolayers than non-zoonotic strain GD-0088 without disrupting the monolayer’s barrier function (A) Enteroid monolayers were apically infected (MOI 50) with *S. suis* strain BM407 (SS2CC1), 861160 (SS2CC20) or GD-0088 (SS9CC16). Translocation events were recorded every 2 h by plating the basolateral medium and the frequency of translocation, expressed as the number of monolayers in which translocation occurred at 2, 4 and 6 h post infection, relative to the total number of monolayers infected, was plotted. Data were obtained from at least 10 monolayers per strain in at least 3 independent experiments. Statistical difference was determined using a log rank (Mantel-cox) test with a Bonferroni correction for multiple testing, error bars show SE and ∗p < 0.05. (B) The barrier function of the enteroid monolayer was assessed during *S. suis* infection by adding FITC-dextran (4 kDa) to the apical compartment and measuring the fluorescence of the basolateral medium. Fluorescence of the basolateral medium was expressed relative to the fluorescence of the apical medium. Statistical difference was determined using a one-way ANOVA with Dunnet’s multiple comparisons test, error bars denote SEM, ∗p < 0.0001. (C) BM407 infected proximal enteroid monolayers were stained for cell nuclei (DAPI, blue), *S. suis* (yellow) and E-cadherin (red, adherens junctions) or (D) ZO-1 (red, tight junctions) and visualized with confocal microscopy. BM407 infected proximal enteroid monolayers were stained for cell nuclei (DAPI, blue), actin (green) and *S. suis* (red), the transwell membrane (TW) was also fluorescent in the DAPI channel. Micrographs are presented as z-stacks (C and E) or maximum projections of a z stack (D).

Adherens junctions and tight junctions present at the cell-cell border are important protein complexes that prevent bacterial translocation via the paracellular route and are often targeted by bacterial pathogens.^37^ Zoonotic *S. suis* was shown to damage the tight junctions in Caco-2 cell monolayers, thereby decreasing the barrier function and permitting bacterial translocation.^9^ To assess if the translocation across the proximal enteroid monolayer was also mediated by the disruption of the enteroid barrier function, proximal derived enteroid monolayers were infected with *S. suis* while monitoring the monolayer barrier function using apically added FITC-dextran (4 kDa; FD4), as previously described for *L. monocytogenes*.^34^ While zoonotic SS2CC1 strain BM407 (80% at 6 hpi) had a higher translocation frequency than the non-zoonotic SS9CC16 strain (30% at 6 hpi) (Figure 2A), permeation of FD4 in the infected monolayers did not change compared to mock infected monolayers. This implied that the monolayer retained its barrier function during *S. suis* translocation and that translocation was not a result of loss of barrier integrity (Figure 2B). Immunofluorescent (IF) staining of infected monolayers did not show decreased or relocated tight junctions and adherens junctions compared to uninfected monolayers (Figures 2C and 2D). In addition, no alterations to the cell surface were observed after *S. suis* infection and translocation, by scanning electron microscopy (Figure S2). With IF staining, we captured the zoonotic SS2CC1 strain BM407 below a cell-cell junction of an intact enteroid monolayer inside a pore of the Transwell membrane, (Figure 2E), suggesting a translocation event.

**Figure 3:**
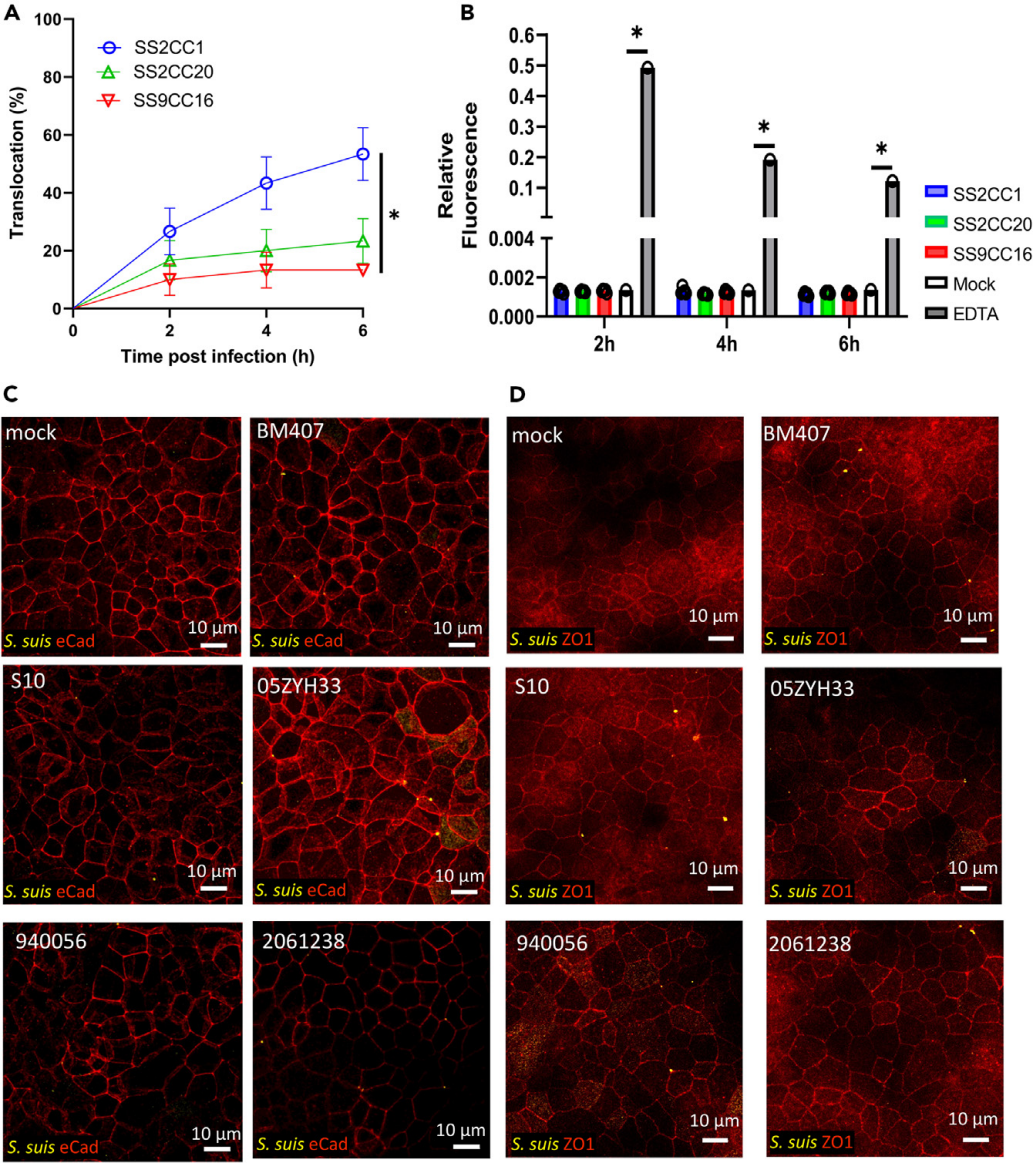
Zoonotic *S. suis* SS2CC1 genotype has a higher translocation frequency across proximal enteroid monolayers than the non-zoonotic SS9CC16 genotype without disrupting the monolayer’s barrier function (A) Enteroid monolayers were apically infected (MOI50) with *S. suis*. Translocation events were recorded every 2 h by plating the basolateral medium and the frequency of translocation, expressed as the number of monolayers in which translocation occurred at 2, 4 and 6 h post infection, relative to the total number of monolayers infected, was plotted. Data were pooled per genotype, with 30 monolayers per genotype. Statistical difference was determined using a log rank (Mantel-cox) test with a Bonferroni correction for multiple testing, error bars show SE and ∗p < 0.01. (B) The barrier function of the enteroid monolayer was assessed during *S. suis* infection by adding FITC-dextran (4 kDa) to the apical compartment and measuring the fluorescence of the basolateral medium. Fluorescence of the basolateral medium was expressed relative to the fluorescence of the apical medium. Statistical difference was determined using a one-way ANOVA with Dunnet’s multiple comparisons test, ∗p < 0.0001. (C) SS2CC1 infected proximal enteroid monolayers were stained for *S. suis* (yellow) and E-cadherin (red, adherens junctions) or (D) ZO-1 (red, tight junctions) and visualized with confocal microscopy. Micrographs are presented as maximum projections of a z stack.

Strains representing *S. suis* CC1 showed an increased median translocation across a differentiated Caco-2 cell monolayer compared to strains from CC16, although translocation varied amongst strains within each CC.^9^ To assess if the observed difference in translocation across proximal enteroid monolayers between *S. suis* strains BM407, 861160, and GD-0088 was representative of the SS2CC1, SS2CC20, and SS9CC16 clonal complexes respectively, in general, we compared five strains from each CC. Strains were selected to capture the diversity in the presence/absence of identified virulence factors within each CC as much as possible (Table S1).^5^ All SS2CC1 strains showed translocation across the proximal enteroid monolayer, but the translocation frequency at 6 hpi ranged from 83% to 17% (Figure S3). Three SS9CC16 strains (GD-0028,GD-0079 and 8067) and two SS2CC20 strains (GD-0119 and 940255) did not show any translocation within 6 hpi (Figure S3). Overall, the translocation frequency of zoonotic SS2CC1 was significantly higher (p = 0.0042) than that of non-zoonotic SS9CC16 (Figure 3A). None of the tested strains increased the enteroid monolayer permeability after infection (Figures 3B; Figure S4). IF staining of the SS2CC1 infected monolayers confirmed that the tight junctions and adherens junctions were unaffected by infection with SS2CC1 (Figures 3C and 3D).

### Zoonotic and non-zoonotic *S. suis* show similar adhesion and invasion

**Figure 4:**
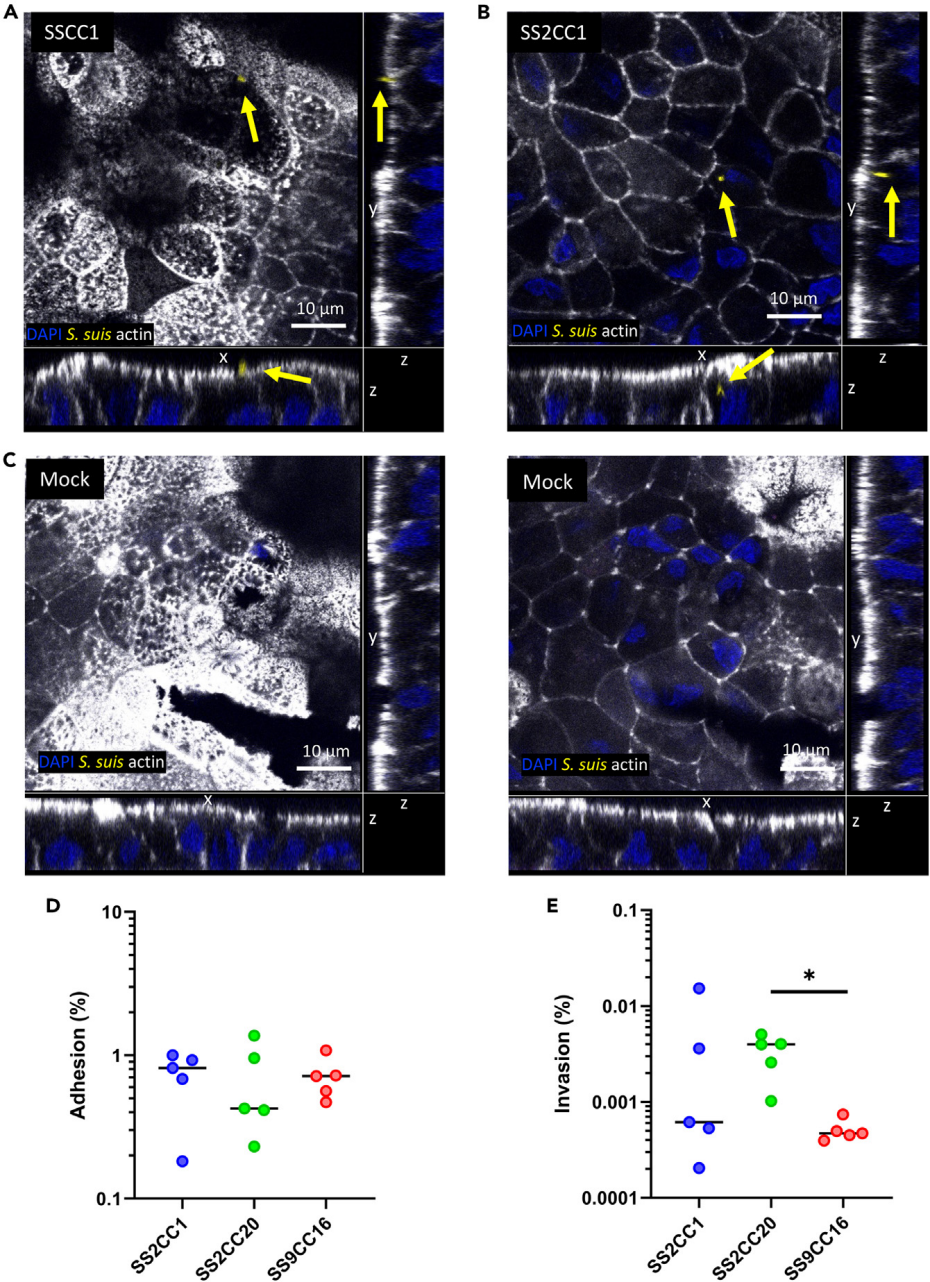
*S. suis* adhesion to proximal enteroid monolayers and invasion of epithelial cells (A–C) *S. suis* infected enteroid monolayer stained was for nuclei (blue), actin (white) and *S. suis* (yellow) and visualized with confocal microscopy. Micrographs show *S. suis* adhesion (A), *S. suis* invasion (B) and a mock infected monolayer (C) and are presented as z stack. (D) Percentage of the inoculum (MOI 10) that adhered to the proximal enteroid monolayer. (E) Percentage of the inoculum (MOI 10) that invaded epithelial cells. (D) Statistical difference was assessed using a one-way ANOVA with Bonferroni correction for multiple testing, (E) or with a Kruskal-Wallis test with Dunn’s multiple correction test, ∗p < 0.05 (E). For D,E, each symbol represents the average per strain and the line denotes the median per genotype.

For multiple foodborne bacterial pathogens, including *S. suis*, the adhesion to the intestinal epithelium or invasion of intestinal epithelial cells has been described as an important step in translocation across the small intestine epithelium.^9^^,^^31^^,^^38^^,^^39^ *S. suis* adhesion to proximal enteroid monolayers and invasion of enteroid epithelial cells were evaluated by IF staining. Both adhesion and invasion events could be observed in the infected enteroid monolayers (Figures 4A–4C). The adhesion and invasion capabilities of the SS2CC1, SS2CC20, and SS9CC16 genotypes were quantified using enteroid monolayers. While some zoonotic strains (05ZYH33, GD-0001, and 2032008) showed 3 to 5-fold lower adhesion to the enteroid monolayer compared to the non-zoonotic GD-0028 strain (Figure S5A), overall the zoonotic and non-zoonotic genotypes showed a similar adhesion (Figure 4D). Most zoonotic strains showed a 10 times higher invasion of epithelial cells than the non-zoonotic strains from SS9CC16 (Figure S5B), while in a comparison across genotypes, only zoonotic SS2CC20 showed significantly increased invasion compared to the non-zoonotic SS9CC16 (Figure 4E).

### Gb3-positive cells are present in proximal enteroid monolayer

**Figure 5:**
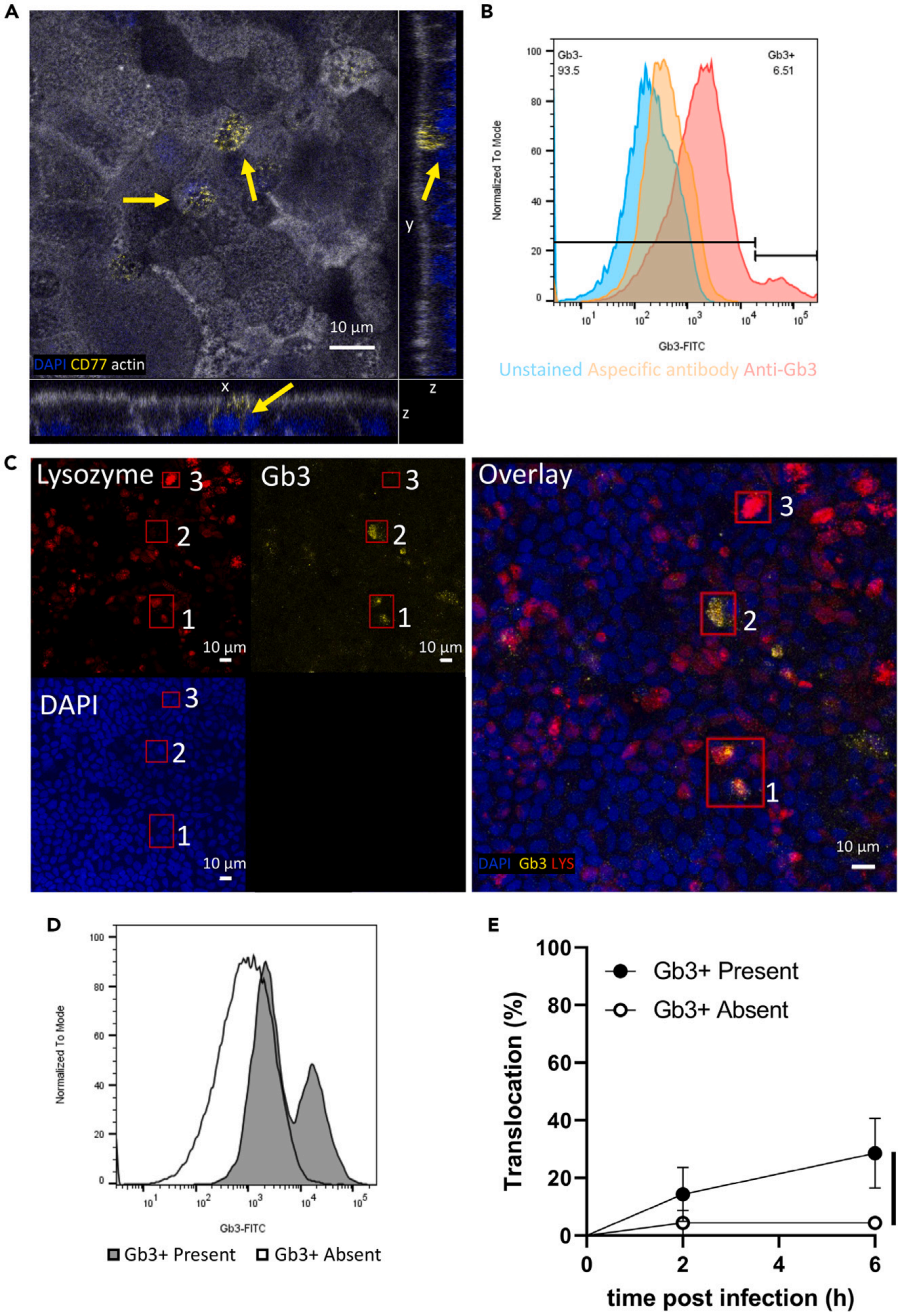
The presence of Gb3-positive cells in proximal enteroid monolayers increases zoonotic SS2CC1 strain 05ZYH33 translocation frequency (A) Monolayer stained for nuclei (blue), actin (white) and Gb3 (yellow) and visualized with confocal microscopy. Control without anti-Gb3 antibody is in supplemental material (Figure S6A). Micrographs are presented as z stack, Gb3-positive cells are indicated by yellow arrow. (B) Presence of Gb3-positive cells in monolayers was assessed by flow cytometry, goat anti-rat IgG Alexa Fluor 488 staining was included as aspecific antibody control. (C) Proximal enteroid monolayer stained for nuclei (blue), Gb3 (yellow) and Lysozyme (red) and visualized with confocal microscopy. Control without primary antibody is in supplemental material (Figure S6B). Micrographs are presented as maximum projections of a z stack. (D) Gb3-positive cell detection by flow cytometry. (E) Monolayers were apically infected (MOI50) with 05ZYH33 WT, translocation events were recorded every 2 h by plating the basolateral medium and the frequency of translocation, expressed as the number of monolayers in which translocation occurred at 2, 4 and 6 h post infection, relative to the total number of monolayers infected, was plotted. Data were obtained from 8 to 10 monolayers. Statistical difference was determined using a log rank (Mantel-cox) test and error bars show SE and ∗p < 0.01.

The translocation of zoonotic SS2CC1 *S. suis* across Caco-2 cell and hCMEC/D3 cell monolayers was previously shown to be mediated by the human glycolipid receptor Gb3.^10^^,^^11^^,^^13^ Thus, we aimed to assess the presence of Gb3-positive cells in the proximal enteroid monolayer, as these Gb3-positive cells could potentially play a role in *S. suis* translocation across the enteroid monolayer. The presence of Gb3 and the transcript of the Gb3 synthase (A4GALT) were recently demonstrated in a pluripotent stem cell derived intestinal organoid.^19^ Similarly, we detected the expression of the *A4GALT* gene in the proximal enteroid monolayer, by RT-PCR (Figure S6) and Gb3-positive cells were identified by IF staining (Figure 5A) and flow cytometry (Figure 5B). Gb3 expression in small intestine epithelial tissue was previously found to be constricted to the crypts, and it has been suggested that Gb3 is expressed by Paneth cells.^17^ To assess if Paneth cells express Gb3 in our model, we stained the enteroid monolayer for Gb3 and lysozyme to identify Paneth cells. The Gb3 staining showed some overlap with the lysozyme staining (Figure 5C box 1), but cells positive for either Gb3 or lysozyme were identified as well (Figure 5C box2 and box3 respectively). In the lysozyme positive cells, the fluorescence intensity of the lysozyme staining was relatively lower in cells that were Gb3-positive than observed for cells that were Gb3-negative (Figure S6C). Despite identical culture conditions, Gb3-positive cells were only present in 4 out of 9 enteroid monolayer culture as detected by flow cytometry (Figure 5C), which was similar to our observation of the IF staining for Gb3 in 2 monolayers of these 9 cultures (Figure S8A). All enteroid monolayers with Gb3-positive cells seem to have a lower TEER value than most enteroid monolayers without Gb3-positive cells, although enteroid monolayers without Gb3-positive cells could have similar low TEER values (Figure S8B). The presence or absence of Gb3-positive cells did not correlate with the passage number of the enteroid cultures (Figure S8C).

### Presence of Gb3-positive cells in the proximal enteroid monolayer increases the *S. suis* translocation frequency

To assess the potential role of Gb3 in the translocation of zoonotic SS2CC1 across the human enteroid monolayers, we used the variation in the presence of Gb3-positive cells in the enteroid monolayers as described above. We studied the role of Gb3-positive cells using the zoonotic SS2CC1 strain 05ZYH33 that was isolated from a diseased patient.^40^ SadP was shown to contribute to 05ZYH33 translocation across an immortalized human cerebral microvascular endothelial cell-line (hCMEC/D3) monolayer by increasing the monolayer permeability in a Gb3 dependent manner.^41^ We evaluated the translocation frequency of 05ZYH33 in nine enteroid monolayer cultures, with two replicates per culture, of which four cultures contained Gb3-positive cells and five cultures did not contain Gb3-positive cells, as determined by flow cytometry. Strain 05ZYH33 translocation frequency was higher (p = 0.044) across enteroid monolayers that contained Gb3-positive cells (29% at 6 hpi) than across monolayers that lacked Gb3-positive cells (4% at 6 hpi) (Figure 5E).

### Gb3-positive dependent translocation can occur independent of streptococcal adhesin protein and suilysin

**Figure 6:**
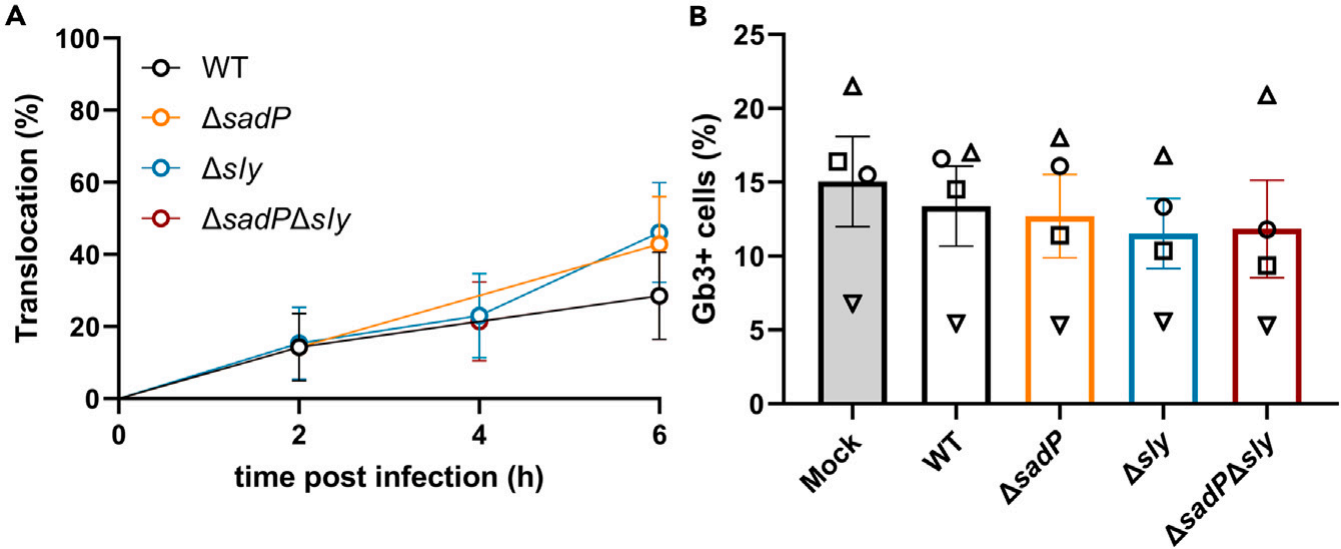
Zoonotic SS2CC1 strain 05ZYH33 translocation across proximal enteroid monolayer does not depend on *sadP* and/or *sly* (A) Gb3-positive cells containing monolayers were apically infected (MOI50) with 05ZYH33 WT, Δ*sadP*, Δ*sly* and Δ*sadP*Δ*sly* and translocation events were recorded every 2 h by plating the basolateral medium and the frequency of translocation, expressed as the number of monolayers in which translocation occurred at 2, 4 and 6 h post infection, relative to the total number of monolayers infected, was plotted. Data were obtained from 8 monolayers per strain. Error bars show SE. Data were obtained simultaneously with data from Figure 5E and thus shows same WT data. (B) Quantification of Gb3-positive cells in a proximal enteroid monolayer was assessed by flow cytometry after 6 h of *S. suis* or mock infection. Each symbol is the average of two monolayers within one experiment, similar symbols indicate data acquired in one experiment. Error bars denote SEM.

The SadP of CC1 is cell wall anchored and binds to the galactose(α1-4)galactose terminal glycan part of Gb3.^10^^,^^11^^,^^42^ In a glycan array, the *S. suis* cholesterol dependent cytolysin named suilysin (Sly) showed high binding to galactose(α1-4)galactose(β1-4)glucose,^43^ which is identical to the glycan structural part of Gb3.^14^ To evaluate the role of SadP and Sly in the translocation of zoonotic SS2CC1 *S. suis* across the enteroid monolayer, we constructed Δ*sadP*, Δ*sly,* and Δ*sadP*Δ*sly* mutants in the strain 05ZYH33. We assessed the translocation frequency of 05ZYH33 WT and KO mutants in the same nine monolayer infection experiments used to study the role of Gb3-positive cells in *S. suis* translocation described above. All mutants were able to translocate across the proximal enteroid monolayer that contained Gb3-positive cells at a similar translocation frequency as WT (Figure 6A). In contrast, in the absence of Gb3-positive cells in the monolayer, the mutants showed no translocation across the proximal enteroid monolayer (Figure S9). To assess if *sadP* and/or *sly* affect the percentage of Gb3-positive cells in the monolayer, the cells of infected monolayers were stained for Gb3 at 6 hpi. None of the monolayers showed a reduction in Gb3-positive cells after infection (Figure 6B).

## Discussion

The emergence of human enteroids allows the study of the initial steps in the pathogenesis of foodborne pathogens in a model that mimics the human small intestine epithelium more precisely than previously used cell line models.^32^^,^^33^ In contrast to human intestinal explants, human enteroids can be subcultured allowing for a multitude of experiments from a single tissue sample.^28^ When studying zoonotic pathogens, animal models such as commonly used pig and mouse models are not appropriate to study the pathogenesis of zoonoses as key proteins or receptors can be host dependent as seen for example in the case of *L. monocytogenes* and human E-cadherin.^44^ Here, we used a human enteroid model to study the translocation of the zoonotic pathogen *S. suis* across the human small intestine epithelium.

Zoonotic *S. suis* infections are predominantly caused by SS2, but these strains can have different genetic backgrounds such as CC1, CC20, CC25 and CC28.^3^ Contrary to SS2CC1, the translocation frequency across a human enteroid monolayer of zoonotic SS2CC20 did not significantly differ from SS9CC16, and two SS2CC20 strains did not show any translocation at all, indicating strain-to-strain variation in translocation within the CC. Nevertheless, we propose that the ability to translocate across the intestinal epithelium is more associated with genetic background than with serotype, which was also observed in translocation experiments using Caco-2 cells.^9^ The presumed routes of human infections by zoonotic CC1 and CC20 strains correlate with the difference in translocation ability between these genotypes. Zoonotic infections by SS2CC1, especially in Southeast Asia, have been linked to the consumption of raw and undercooked pig products, arguing for an infection route via the gastrointestinal tract.^45^^,^^46^ Zoonotic SS2CC20 infections have only been described in the Netherlands, where *S. suis* infection is an occupational hazard for which it appears most likely that *S. suis* enters the bloodstream after direct infection via skin lesions.^5^^,^^46^^,^^47^

The proximal and distal small intestine differ in cell composition, which is reflected by the enteroid monolayers generated from these tissue segments.^34^^,^^48^ For zoonotic *S. suis*, the translocation was higher across proximal derived enteroid monolayers than distal derived enteroid monolayers. In contrast, using highly similar enteroid monolayers, the translocation of *L. monocytogenes* was higher in distal derived monolayers than proximal derived monolayers.^34^ Together, this suggests that the observed difference in translocation between proximal and distal derived enteroid monolayers is the result of specific host-pathogen interactions. Translocation of different *S. suis* genotypes in models of other sections of the gut, such as the colon, was not evaluated.

Our data suggests that Gb3-positive cells are indeed present in the human small intestine epithelium, although the frequency of Gb3-positive cells varied. Culture conditions yet unknown may influence the development of Gb3-positive cells and/or affect the Gb3 expression in these cells during enteroid monolayer culture. The presence of Gb3-positive cells in the human small intestine and colon epithelium has been disputed. Gb3-positive cells were suggested to be present in the colon epithelium despite being unable to detect the Gb3-positive cells by IF staining of two tissue samples.^18^ The presence of Gb3-positive cells in small intestine epithelium was shown in IF staining of healthy duodenal tissue cryosections and the same study reported that none of the 17 colonic epithelium tissue samples stained positive for Gb3.^17^ However, similar staining of intestinal tissue, including duodenal tissue, did not detect Gb3-positive cells in the intestine epithelium,^16^^,^^18^ suggesting that it is challenging to successfully stain for and detect the presence of Gb3. Here, the proportion of Gb3-positive cells in enteroid monolayers appeared lower in the IF staining than in the flow cytometry. Gb3 is a glycolipid that is inserted into a cell membrane, and potentially Gb3 is partially lost during the IF staining procedure, for example due to multiple washing steps, the use of TWEEN 20, or the fixation prior to staining. In the flow cytometry staining approach, cells are first stained and then fixed, and only washed twice in total and not exposed to TWEEN 20. Therefore, these differences between IF staining and flow cytometry could explain the observed discrepancy in Gb3+ cell abundance between the two approaches.

Human pluripotent stem cell derived intestinal organoids were previously shown to have Gb3-positive cells, but the cell type expressing Gb3 was not characterized.^19^ Here, Gb3-positive cells were detected in differentiated proximal enteroid monolayers by IF staining and flow cytometry. In the duodenal tissue, the Gb3-positive cells were reported within the crypts and showed co-localization with a lysozyme staining which is a marker often used to identify Paneth cells.^17^ Hence, it was suggested that Paneth cells are Gb3-positive. In our enteroid monolayer, a subset of the Gb3-positive cells stained positive for lysozyme as well suggesting that Gb3-positive cells are Paneth cells. However, not all lysozyme positive cells were Gb3-positive. In adult human intestinal tissue, lysozyme expression was found not to be restricted to Paneth cells and even higher expressed by BEST4+ cells and Follicle-Associated Epithelium cells, including M cells, and to a lesser extent expressed by Tuft cells.^48^^,^^49^ In adult intestinal tissue, Paneth cells increase in abundance distally.^50^ In our organoid model, the translocation of zoonotic *S. suis* was decreased in distal derived enteroid monolayers compared to proximal derived enteroid monolayers, and we demonstrated that translocation correlates with the presence of Gb3-positive cells. The inverse correlation of Paneth cells abundance and translocation suggests that Gb3-positive cells in our enteroid model are not Paneth cells. Gb3-positive cells identified as Paneth cells have only been detected in duodenal tissue,^17^ thus Gb3-positive Paneth cells could be limited to duodenal tissue and enteroids derived from this tissue. Whilst it is not possible to distinguish between duodenal and jejunal epithelium in our model, this distribution could explain the decreased translocation in the enteroids derived from the distal small intestine.

The presence of Gb3-positive cells in the enteroid monolayer was shown to correlate with the translocation of zoonotic *S. suis*. Although the correlation between translocation and the presence of Gb3-positive cells should be confirmed with other strains from strains from different genetic backgrounds. Enteroid monolayers with Gb3-positive cells tended to have a lower TEER value, potentially indicating a priming effect of Gb3-positive cells for easier translocation, which could explain the observed correlation. However, enteroid monolayers without Gb3-positive cells could have similar low TEER values. Besides the observed correlation, host cell receptors other than Gb3 are likely also involved in the translocation. Other human (enteric) pathogens such as Shiga toxin producing *Escherichia coli* or *Shigella dysenteriae,* uropathogenic *E. coli*, *Bacillus cereus,* and *Pseudomonas aeruginosa* also interact with Gb3, suggesting that Gb3 targeting by human pathogens is an example of convergent evolution.^19^^,^^51^^,^^52^^,^^53^^,^^54^^,^^55^ However, the effect of Gb3 binding by these pathogens (produced toxins) differs, from adhesion, invasion, to inducing cell death. Shiga toxin binding to Gb3 induces necrotic and apoptotic cell death,^19^ *B. cereus* uses a flagellin to bind to Gb3^51^ and *P. aeruginosa* binds to Gb3 with its bacterial surface lectin LecA, which triggers a lipid zipper dependent engulfment of the bacteria leading to host cell invasion.^52^^,^^56^ The Gb3 binding by these pathogens is influenced by the lipid tail of Gb3 and the composition of the lipid bilayer in which Gb3 is embedded,^57^ thus the specificity of a bacterial virulence factor for Gb3 is not solely dependent on the glycan structural part of Gb3. Thus, although the presence of Gb3-positive cells correlates with zoonotic *S. suis* translocation across the enteroid monolayer, the mechanism by which interaction with Gb3 contributes to translocation is difficult to predict based on comparisons with other Gb3 targeting pathogens. Further efforts to verify the presence of Gb3-positive cells in the crypts of small intestine epithelium and to elucidate the cell type expressing Gb3 in intestinal tissue and enteroid cultures are needed to better understand the pathogenesis of infection caused by *S. suis* and other pathogens that show interaction with Gb3-positive cells.

In a Caco-2 model, the reported median percentage of invasion and adhesion was respectively a 5-or 10-fold higher than we found in our enteroid monolayers.^9^ This difference could be attributed to procedural differences such as the additional two washing steps. However, similar levels of invasion and adhesion as we observed in the enteroid monolayer model have been reported in a NPTr porcine tracheal epithelial cell line model.^58^ Moreover, adhesion and invasion were shown to be cell-line dependent.^59^

*S. suis* adhesion to Caco-2 cells was previously shown to be mediated by Gb3 accessibility.^10^ We observed a similar adhesion to the enteroid monolayer by SS2CC1 and SS9CC16, which could be explained by the low abundance of Gb3-positive cells in the enteroid monolayer. In contrast to the enteroid monolayer, all Caco-2 cells express the Gb3 synthase gene *A4GALT* and cells show a strong staining for Gb3 on their surface.^60^^,^^61^ The translocation of zoonotic *S. suis* SS2CC1 across hCMEC/D3 and Caco-2 cell monolayers decreased in Δ*sadP* strains, but was not completely abolished.^10^^,^^13^ Similarly, the Δ*sadP* mutant could still translocate across the proximal enteroid monolayer. In Southeast Asia, most *S. suis* infections are foodborne, but 24% of the zoonotic *S. suis* isolates included in a systematic review lacked *sadP*.^62^ Moreover, in 2021, a *sadP (fhb)* deficient strain caused a foodborne *S. suis* outbreak in Thailand,^63^ thus *sadP* seems not essential for *S. suis* to cause foodborne infections. However, the Southeast Asian isolates described in these two studies all have *sly*.^62^^,^^63^ Despite this association, we observed no reduction in translocation in a Δ*sly* mutant, as was also shown in a Caco-2 model.^9^ Both Sly and SadP can bind to Gb3,^10^^,^^11^^,^^42^^,^^43^ but the data suggests that other *S. suis* genes are also involved in the Gb3 dependent translocation across the human small intestine epithelium.

In general, enteric pathogens can cross the epithelial barrier by two mechanisms, paracellular (between cells) or transcellular (through cells),^64^ and some pathogens such as *L. monocytogenes* show both paracellular and transcellular translocation.^64^ Pathogens that target Gb3 were shown to be able to use both mechanisms,^19^^,^^51^^,^^52^^,^^53^^,^^54^^,^^55^ thus the Gb3 targeting by *S. suis* does not seem to favor one of the two mechanisms. The invasion of epithelial cells is the first step in transcellular translocation.^64^ Due to the low invasion of epithelial cells by zoonotic *S. suis* from SS2CC1, we speculate that translocation does not occur transcellular. Indeed, the captured translocation event of zoonotic *S. suis* implies a paracellular translocation mechanism. The paracellular translocation is not preceded by loss of barrier function by the enteroid monolayer, nor by major rearrangements of the adherens junctions and tight junctions, thus suggesting a transient opening of the cell junctions. A similar transient opening of the cell junctions enabling the translocation across epithelial monolayers has been described for *Streptococcus agalactiae*^65^ and *Streptococcus gallolyticus* subsp. *Gallolyticus*.^66^ Taken together, we speculate that zoonotic SS2CC1 *S. suis* translocates across the enteroid monolayer via the paracellular route with a transient opening of the cell junctions, which is mediated by its interaction with Gb3-positive cells.

Human enteroid monolayers are a considerably improved model for studying enteric pathogens compared to immortalized cell lines but still lack components that intestinal tissue has, including crypt-villus structure, microflora, mesenchyme, mechanical flow and immune components such as intestinal intraepithelial lymphocytes.^67^^,^^68^ Human *S. suis* infections are almost exclusively reported in adults,^2^ thus an adult enteroid model would be preferred to study zoonotic *S. suis* intestinal translocation. The monolayers we used to study *S. suis* translocation were generated from fetal derived enteroids, which have been used to study enteric pathogens by others,^69^^,^^70^ but show transcriptional differences compared to adult derived enteroids.^71^ However, the transcriptional profile of late fetal enteroids, that are also used in this study, was more similar to that of adult enteroids than early fetal enteroids.^71^ Additionally, we used differentiated enteroid monolayers, while the early fetal, late fetal, and adult enteroid transcriptomic profiling was performed on undifferentiated 3D enteroids that likely show a less mature phenotype.^34^^,^^71^ Small intestine organoids and epithelial monolayers can also be generated from induced pluripotent stem cells, which have the advantage not to rely on tissue availability.^67^ The disadvantage of pluripotent stem cell derived organoids is their more fetal than adult transcriptional profile.^67^ Despite the less mature phenotype of pluripotent stem cell or fetal derived enteroids, both allow for studying enteric pathogens likely yielding similar results as those obtained from adult derived enteroids. For example, a study on *S. enterica* serovar Typhimurium infection and the role of secretory IgA used monolayers generated from pluripotent stem cell and adult tissue derived enteroids, and found that both monolayers gave similar findings, although the magnitude of the outcome differed.^72^ Thus, despite the less mature phenotype, pluripotent stem cells or fetal derived enteroids can be useful models to study enteric pathogens.

Despite these limitations, we show that the zoonotic *S. suis* SS2CC1 genotype can translocate across a human enteroid monolayer. Zoonotic SS2CC1 translocated at a higher frequency across human enteroid monolayers than non-zoonotic *S. suis* SS9CC16, that is virulent in pigs, while these genotypes did not differ in adhesion and invasion. Lastly, *S. suis* translocated across the enteroid monolayer without compromising the epithelial barrier function. We confirmed the presence of Gb3-positive cells in human enteroids and found that translocation correlates with the presence of these Gb3-positive cells within the enteroid monolayer.

### Limitation of this study

In the present study, zoonotic SS2CC1 *S. suis* shows increased translocation across a proximal small intestine enteroid monolayer compared to non-zoonotic SS9CC16. Which genes, which are present within SS2CC1 and absent in SS9CC16, are responsible for this difference in translocation frequency remains unknown. Additionally, we have only tested a limited amount of *S. suis* strains per CC, so there could be other mechanisms of translocation in untested strains. While a correlation between the presence of Gb3-positive cells and translocation of zoonotic *S. suis* was observed, the underlying mechanism of translocation was not explored yet, although data suggest a paracellular route. The translocation frequency of zoonotic SS2CC1 was lower in distal than proximal derived enteroids, which could be due to the respectively presence or absence of Gb3-positive cells. However, we do not know if Gb3-positive cells are present within distal derived enteroids. We demonstrated the presence of Gb3-positive cells in proximal derived enteroids, but we were unable to define which cell type is Gb3-positive.

## STAR★Methods

### Key resources table

**Table:** 

REAGENT or RESOURCE	SOURCE	IDENTIFIER
**Antibodies**		

Monoclonal Rat IgG Anti-eCadherin	eBioscience	14-3249-82; RRID: AB_1210458
Monoclonal Rat IgG Anti-ZO-1	Santa Cruz Biotech	R40.76; RRID: AB_628459
Polyclonal Rabbit IgG anti-Lysozyme	Invitrogen	PA5-16668; RRID: AB_10984852
Rabbit serum anti- *S. suis* serotype 2	Statens Serum Institut	22282
Purified mouse IgM anti-human CD77	Biolegend	357102; RRID: AB_2561847
Purified mouse IgM anti-human CD77, FITC-labelled	Biolegend	357103; RRID: AB_2562160
Goat anti-rat IgG (H + L)Alexa Fluor 488	Invitrogen	A-11006; RRID: AB_2534074
Phalloidin CruzFluor™ 488 Conjugate	Santa Cruz Biotech	sc-363791
Goat anti-Rabbit IgG (H + L) Cross-Adsorbed Secondary Antibody, Alexa Fluor 555	Invitrogen	A-21428; RRID: AB_2535849
Goat anti-Mouse IgM (Heavy chain) Cross-Adsorbed Secondary Antibody, Alexa Fluor 555	Invitrogen	A-21426; RRID: AB_2535847
Goat anti-rabbit IgG (H + L)Alexa Fluor 633	Invitrogen	A-21070; RRID: AB_2535731
Goat anti-Rat IgG (H + L) Cross-Adsorbed Secondary Antibody, Alexa Fluor 647	Invitrogen	A-21247; RRID: AB_141778
DAPI	Invitrogen	D1306

**Bacterial and virus strains**		

See Table S2	N/A	N/A

**Chemicals, peptides, and recombinant proteins**		

Human Intesticult Growth Medium	STEMCELL Technologies	Cat#06010
Penicillin–streptomycin	Gibco	15140122
Matrigel	Corning	356231
CryoStor CS10	STEMCELL Technologies	Cat#07930
Y-27632	STEMCELL Technologies	Cat#72302
DMEM/F12	Gibco	12634010
HEPES	Gibco	15630056
PET-membrane inserts (0.33 cm^2^, 3.0 μm pore size)	VWR	734–2747
TRYPLE	Gibco	12605010
FITC-Dextran 4kDa	Sigma	FD4-100MG

**Critical commercial assays**		

Phusion Hot Start II DNA polymerase kit	ThermoScientific	F549L

**Oligonucleotides**		

See Tables S3 and S5	Sigma	N/A

**Software and algorithms**		

LasX	Leica	N/A
GraphPad Prism	GraphPad	N/A
Flowjo	Flowjo	N/A

### Resource availability

#### Lead contact

Further information and requests for resources and reagents should be directed to and will be fulfilled by the lead contact, Dr. Constance Schultsz (MD) (c.schultsz@aighd.org).

#### Materials availability

This study did not generate new unique materials or reagents.

#### Data and code availability

• Data - The whole genome sequencing data used to confirm the successful mutant generation are publicly available as of the date of publication, the data can be downloaded from ENA project: PRJEB71334.
• Code - This paper does not report original code.
• Other - Any additional information required to reanalyze the data reported in this paper is available from the lead contact upon request.

### Experimental model and study participant details

#### Ethics statement

Human fetal intestinal tissue (gestational age 18–20 weeks) was obtained by the HIS Mouse Facility of the Amsterdam UMC from the Bloemenhove clinic (Heemstede, the Netherlands), with a written informed consent obtained from all donors for the use of the material for research purposes. These informed consents are kept together with the medical record of the donor by the clinic. Tissues were obtained with approval of the ethical committee of the Amsterdam UMC, together with approval of the experimental procedures by the HIS Mouse Facility (Amsterdam UMC).^34^ All methods were performed in accordance with the relevant guidelines and regulations, as stated in the Amsterdam UMC Research Code.

#### Enteroid culture

Human enteroid cultures were generated from fetal intestinal tissue and maintained as previously described.^34^ Shortly, crypts from the proximal (duodenum, jejunum) or distal (ileum) small intestine were isolated from human fetal tissue. Duodenal and jejunal sections were pooled because we were unable to separate these sections based on macroscopical features during dissection. The crypts were cultured in matrigel (Corning) droplets as described by Sato et al.^30^ Crypts/enteroids were cultured in three 10 μL matrigel droplets per well (24-wells plate) overlayed with Human Intesticult Growth Medium (STEMCELL Technologies) supplement with 100 U-mg/mL penicillin–streptomycin and incubated at 37°C, 5% CO_2_. Medium was refreshed every 2–3 days and enteroids were passaged by mechanical dissociation^30^ or enzymatic dissociation (see enteroid translocation assay) every 6–10 days. Enteroids were resuspended in 1 mL of CryoStor CS10 (STEMCELL Technologies) for long term storage at −140°C, after which cultures could be restarted by resuspending washed organoids in matrigel as described above.

#### Enteroid monolayer culture

Enteroid monolayers were cultured on inserts (PET-membrane, 0.33 cm^2^, 3.0 μm pore size; VWR) as previously described.^34^ Briefly, membranes were coated with 100 μL 0.01% (v/v) acetic acid with 20 μg/mL collagen type I (rat tail, Ibidi) for 1 h at room temperature (RT) and washed twice with PBS. Enteroids were collected and dissociated into a single cell suspension by 10 min incubation at 37°C in TrypLE (Gibco). Cells were washed to remove TrypLE and the volume of the cell suspension was adjusted to reach a concentration of 10^6^ cells/mL of which 100 μL was seeded per insert. Cells were cultured in Human Intesticult Growth Medium containing 10 μM Y-27632 (STEMCELL Technologies) and 100 U-mg/mL penicillin–streptomycin for 3 days, after which Y-27632 was removed. After 7 days, monolayers were differentiated for 7 days by replacing the medium with a 1:1 mixture of Human Intesticult Growth Medium component A/Basal medium and advanced DMEM/F12 (Gibco) containing 1x Glutamax (Gibco), 15 mM HEPES (Gibco) named ADF++ supplemented with 100 U-mg/mL penicillin–streptomycin (Gibco). The apical (100 μL) and basolateral medium (600 μL) were refreshed every 3–4 days. Monolayer formation was monitored by regularly measuring the TEER.

For the adhesion and invasion assay enteroid monolayers were cultured in 48 wells plates. Wells were coated (1 h, RT) with 800 μL of 25 μg/mL collagen type I in 0.01% (v/v) acetic acid and washed twice with PBS. Wells were seeded with 150.000 cells in 250 μL in Human Intesticult Growth Medium containing 10 μM Y-27632 for 2–3 days. Upon reaching confluence, monolayers were differentiated for 5 days using the same medium as used for monolayers cultured on inserts and replacing medium every 2–3 days. All cultures, also during infection experiments, were incubated at 37°C, 5% CO_2_.

### Method details

#### Bacterial strains and mutants construction

*S. suis* strains and mutants are listed in the supplemental materials (Table S2). Bacteria were cultured in Todd-Hewitt Broth supplemented with 0.5% yeast extract (THY) or on THY plates at 37°C, supplemented with 200 μg/mL kanamycin or 2 μg/mL erythromycin when required. Mutants were generated using homologues recombination via induced competence and mutator fragments with kanamycin or erythromycin resistance flanked by homologues regions as previously published.^73^ Mutator fragments were amplified from S10Δ*sadP*^10^ and S10Δ*sly*^74^ using the primers listed in the supplemental materials (Table S3) using the Phusion Hot Start II DNA polymerase kit (ThermoScientific) according to manufacturer’s protocol. Successful interruption of target genes in mutants was checked by PCR (Figure S10) and whole genome sequencing (ENA project: PRJEB71334).

#### Enteroid translocation assay

Translocation assay was adapted from a previously published Caco-2 *S. suis* infection experiment.^10^ Differentiated monolayers grown on inserts with a minimal TEER of 250 Ω∗cm^2^ for distal and of 300 Ω∗cm^2^ for proximal enteroids were washed twice with ADF++ to remove antibiotics.^34^ Enteroid monolayers were cultured in ADF++ during the infection experiment. *S. suis* was added to the apical compartment (Multiplicity of Infection [MOI] 50) and after approximately 5 min a 100 μL sample of the basolateral medium was plated. If culture of the sampled basolateral medium was positive for *S. suis*, the barrier function of the monolayer was compromised at the start of the infection experiment and the monolayer was excluded. Monolayers were incubated for 6 h, and every 2 h basolateral medium was sampled and refreshed to prevent bacterial overgrowth. Collected basolateral medium was serially diluted and plated on blood agar plates to quantify CFU. Translocation was defined as present when detecting any count of CFU in the basolateral medium and the frequency of translocation was expressed as a proportion, i.e., the number of monolayers in which translocation occurred relative to the total number of monolayers infected.

The barrier integrity was monitored with a dextran permeability assay, which have been shown to correlate with TEER measurements.^34^ The barrier permeability during the infection experiment was monitored by adding 150 μg FD4 (Sigma) to the apical compartment and using phenol red free ADF++ (Gibco) in the infection experiment. Enteroid monolayers without *S. suis* or monolayers incubated with Hank’s Balanced Salt Solution without Ca^2+^ and Mg^2+^ (Lonza) with 2 mM EDTA were included as controls for barrier permeability. The fluorescence of 10 times diluted apically added FD4 and undiluted basolateral samples were measured in a Synergy H1 plate reader. Barrier permeability was expressed in relative fluorescence of the basolateral medium to the apically added medium and corrected for volume differences.

#### Enteroid adhesion and invasion

Adhesion and invasion assay was adapted from a previously published Caco-2 *S. suis* infection experiments.^10^ Briefly, enteroid monolayers were washed to remove antibiotics and medium was changed to ADF++, before adding *S. suis* resuspended in ADF++ (MOI 10). After 2 h, non-adherent bacteria were removed by washing 5 times with ADF++. For adhesion, cells were lysed with ice-cold demi water and bacterial suspensions were serial diluted in PBS and plated on blood agar plates. For invasion, extracellular bacteria were killed with 100 μg/mL gentamicin and 5 μg/mL penicillin G for 2 h and efficient killing was verified by plating the cell supernatant after 2 h. Antibiotics were removed and cells were washed 3 times with ADF++, before lysing with ice-cold demi water and subsequent plating. CFU of invading bacteria were subtracted from adherent bacteria and the percentage of adhesion and invasion relative to the inoculum was calculated.

#### Immunofluorescence staining and confocal microscopy

Enteroid monolayers were fixed in 4% formaldehyde in PBS for 30 min at RT, then washed twice with PBS and stored in PBS at 4°C until staining. Membranes with enteroid monolayers were cut from inserts using a scalpel. Monolayers were permeabilized and blocked, either by 30 min incubation in PBS with 3% bovine serum albumin (BSA) and 0.1% Triton X-, by 10 min permeabilization in methanol at −20°C followed by washing 3 times with PBS and 30 min blocking in PBS containing 3% BSA or by blocking only in PBS with 3% bovine serum albumin (BSA). Cells were stained with primary antibodies for 2 h at RT, subsequently washed 3 times with PBS for 5 min and stained with secondary antibodies and actin probes for 1 h at RT. Nuclei were stained with DAPI and monolayers were washed 3 times with PBS for 5 min before mounting overnight at RT in ProLong Diamond Antifade Mountant (with DAPI; Invitrogen). Slides were stored at 4°C until image acquisition. All antibodies were diluted in PBS containing 3% BSA with 0.1% TWEEN 20 and are listed in supplemental materials (Table S4). Imaging was performed at the Amsterdam UMC Cellular Imaging Core Facility using a Leica TCSS SP8 X mounted on a Leica DMI6000. Images were processed using LasX (Leica, version 3.7.1) software.

#### Flow cytometry

After washing the monolayer with PBS, cells were detached by 30 min incubation in ADF++ with 1 mg/mL Collagenase D and 100 U/mL DNAse I and subsequent scraping with a 1 μL inoculation loop. Cells were washed with PBS and transferred to a V-bottom plate. Cells were stained in 25 μL for CD77 using FITC-labelled mouse IgM (Table S4) diluted in PBS for 30 min at 4°C, while shaken mildly. Cells were washed twice with PBS and fixed with 4% formaldehyde in PBS for 15 min at RT. Fixed cells were washed with PBS and stored in PBS at 4°C until acquisition (maximum 24 h). Cells were measured on the BD FACSCanto at the Amsterdam UMC Cellular Imaging Core Facility and data were analyzed using Flowjo software (version 10.8.1).

#### Scanning electron microscopy

Monolayers were fixed, dehydrated and coated as described previously^34^ and imaged at the Amsterdam UMC Cellular Imaging Core Facility using a Zeiss Sigma-300 FE scanning electron microscope.

#### RT-PCR

RNA was isolated from two pooled enteroid monolayers using TRIzol (Invitrogen) and the Direct-zol miniprep (Zymo Research) according to manufacturer’s protocol. Genomic DNA was removed with the TURBO DNA free kit (Invitrogen) in presence of SUPERase RNAse inhibitor (Invitrogen). cDNA was made with the Superscript IV first strand synthesis kit (Invitrogen) using oligo(dT)_20_ and random hexamers. Gene expression was assessed by PCR using the cDNA as template, the primers listed in the supplemental material (Table S5) and the GO Taq PCR kit (Promega). Amplicons were visualized on a 1% agarose gel.

### Quantification and statistical analysis

All statical analyses were performed using GraphPad Prism (v9.3.1, GraphPad Software). For the translocation assays, p values indicated in the text are adjusted p values obtained from a log rank (Mantel-cox) test with a Bonferroni correction for multiple testing. All other data showed a normal distribution except for the data from Figure 4E, which had a p < 0.0001 in the Shapiro-Wilk test. Statistical tests and p values are indicated in figure legends.
