# Supplementary material for: Translocation across a human enteroid monolayer by zoonotic Streptococcus suis correlates with the presence of Gb3-positive cells
Source: iScience. 2024 Feb 12;27(3):109178. doi: 10.1016/j.isci.2024.109178 (PMC10909756; doi:10.1016/j.isci.2024.109178)
Supplement: Document S1. Figures S1–S10 and Tables S1–S5 [file mmc1.pdf]

iScience, Volume *27*

Supplemental information

Translocation across a human enteroid monolayer by zoonotic *Streptococcus suis* correlates with the presence of Gb3-positive cells

Thomas J. Roodsant, Kees C.H. van der Ark, and Constance Schultsz

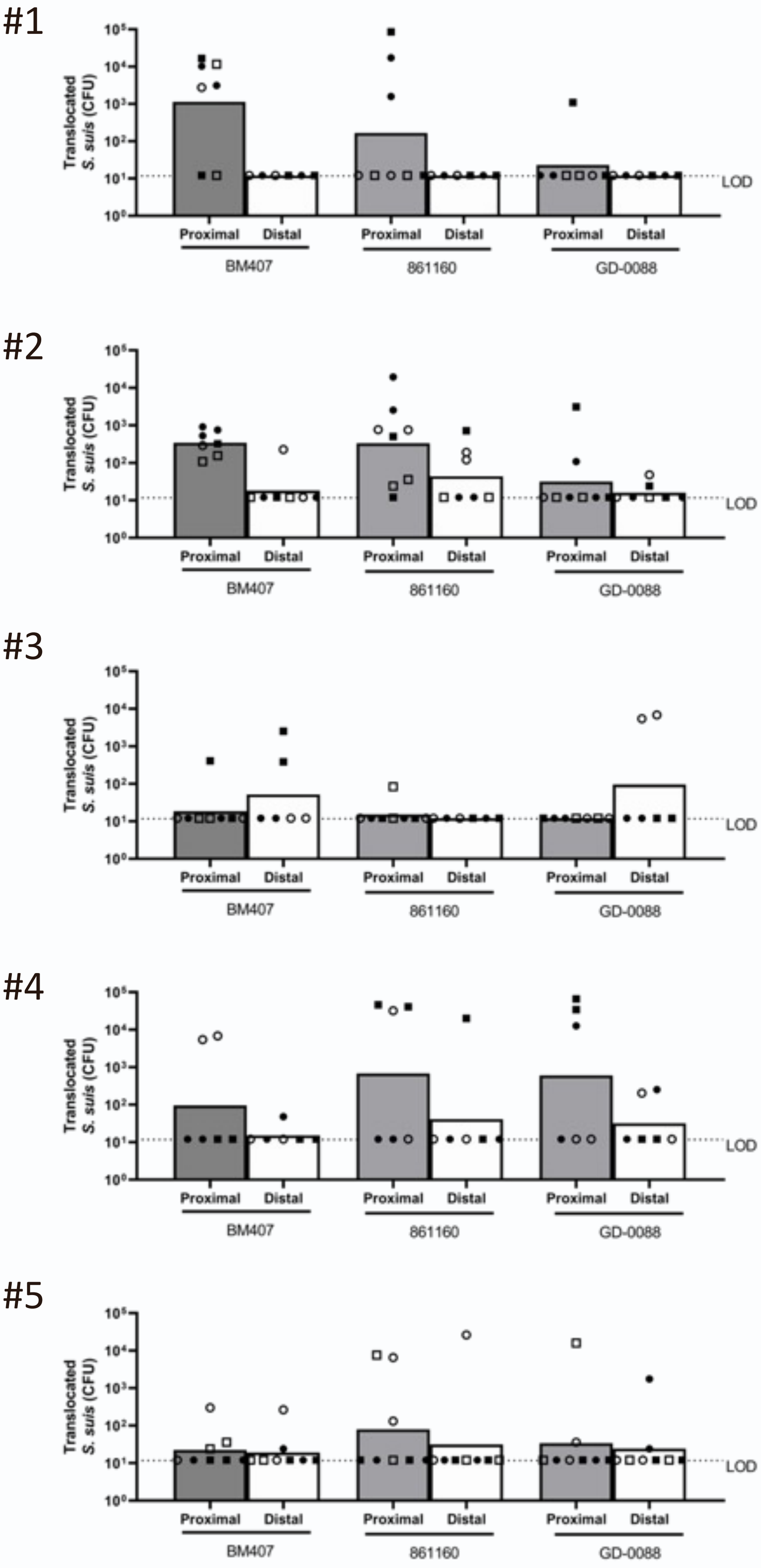

FIG S1 ***S. suis*** **translocation across proximal and distal enteroid monolayers per donor related to Figure 1.** Enteroid monolayers were apically infected (MOI50) with *S. suis* strain BM407 (SS2CC1), 861160 (SS2CC20) or GD-0088 (SS9CC16). Translocation was quantified every 2 h and total number of translocated bacteria after 6 h was plotted. Each symbol represents one monolayer, similar symbols per donor indicate data obtained within the same experiment. Data were generated in at least 3 independent experiments with in total at least 6 infected monolayers per donor. Bar graphs show mean translocation. LOD, limit of detection.

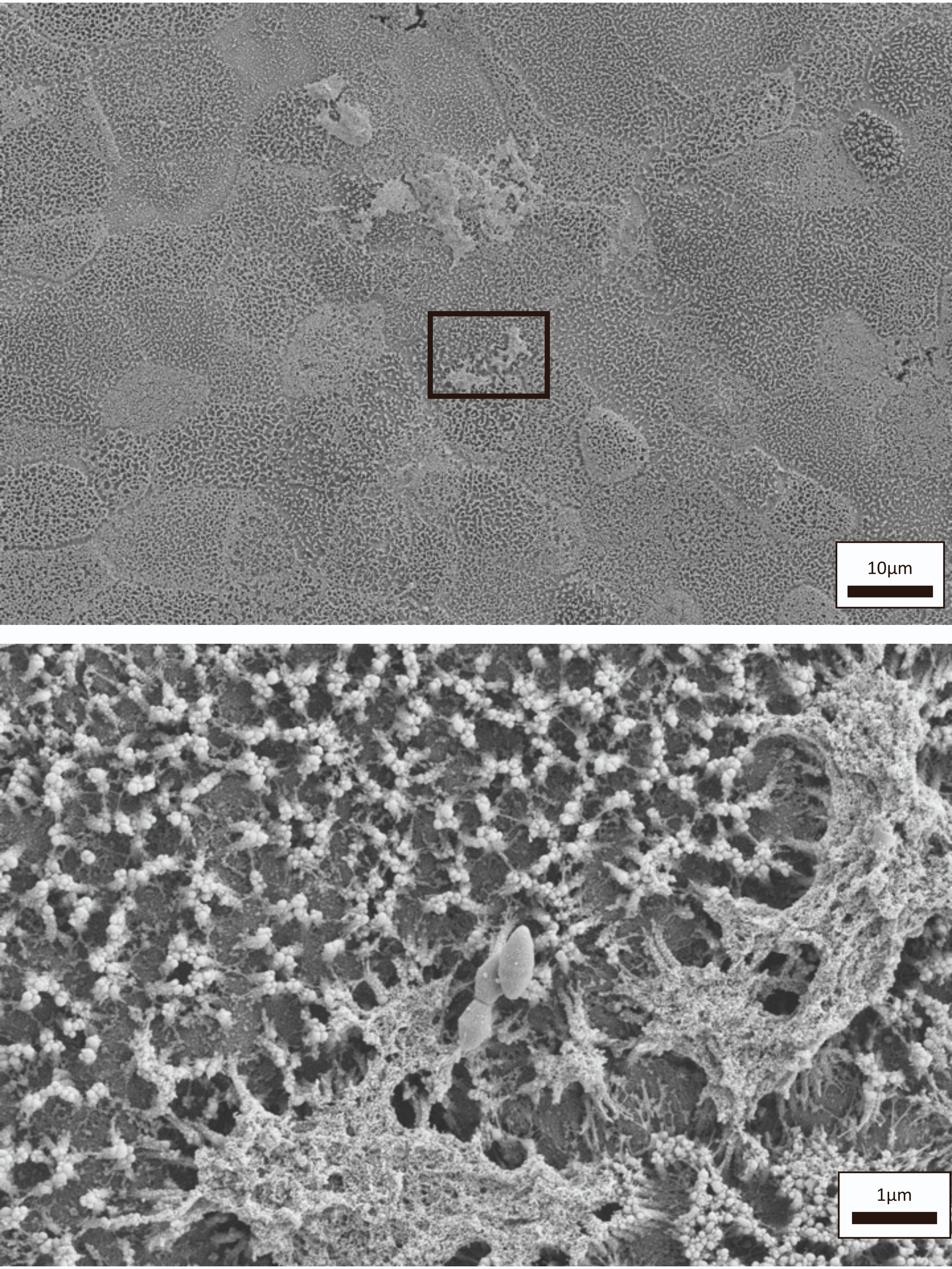

FIG S2 Scanning electron microscopy (SEM) of apical surface of proximal enteroid monolayer after *S. suis* SS2CC1 strain BM407 infection related to Figure 2.

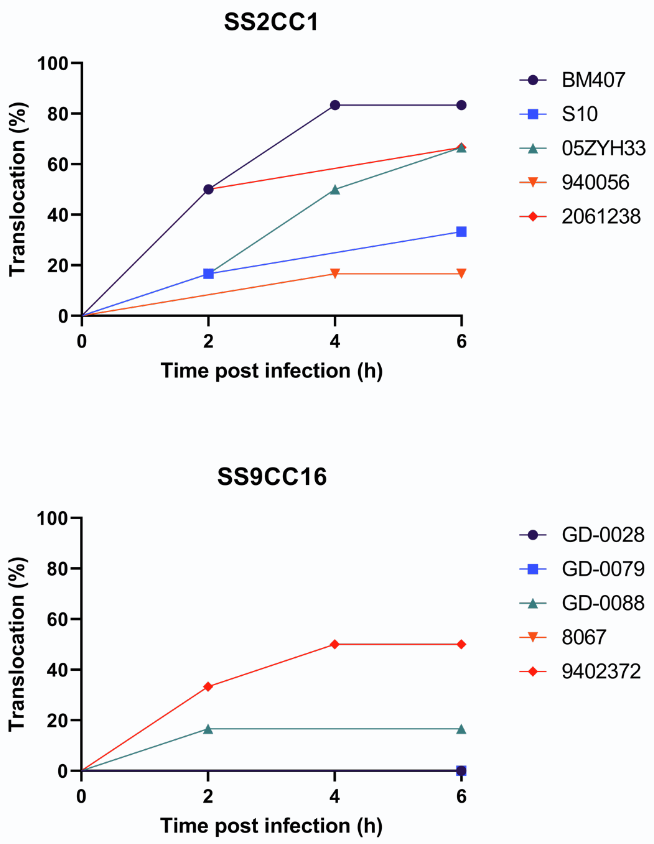

FIG S3 Translocation of SS2CC1, SS2CC20 and SS9C16 *S. suis* strains across proximal enteroid monolayers related to Figure 3. Enteroid monolayers were apically infected (MOI50) with *S. suis* and translocation events were recorded every 2 h by plating the basolateral medium and the frequency of translocation, expressed as the number of monolayers in which translocation occurred at 2 h, 4 h and 6 h post infection, relative to the total number of monolayers infected, was plotted. The percentage of monolayers in which translocation occurred was plotted for each strain. Data per strain was obtained from 6 monolayers in 3 separate experiments.

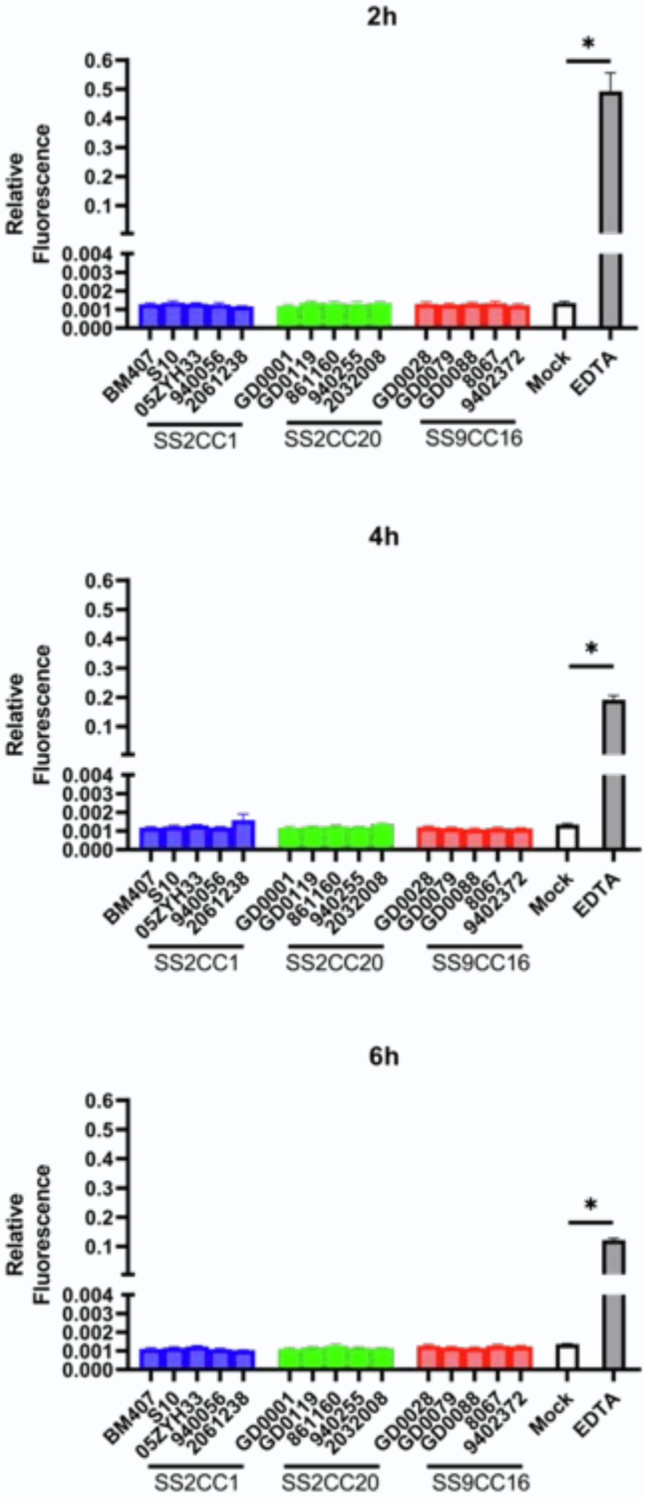

FIG S4 Apical infection of proximal enteroid monolayers with *S. suis* from SS2CC1, SS2CC20 and SS9C16 does not affect barrier permeability related to Figure 3. The barrier function of the enteroid monolayer was assessed during *S. suis* infection by adding FITC-dextran (4kDa) to the apical compartment by measuring the fluorescence of the basolateral medium at 2, 4 and 6 h. Fluorescence of the basolateral medium was expressed relative to the fluorescence of the apical medium. Statistical difference was determined using a one-way ANOVA with Dunnet's multiple comparisons test, *p<0.0001.

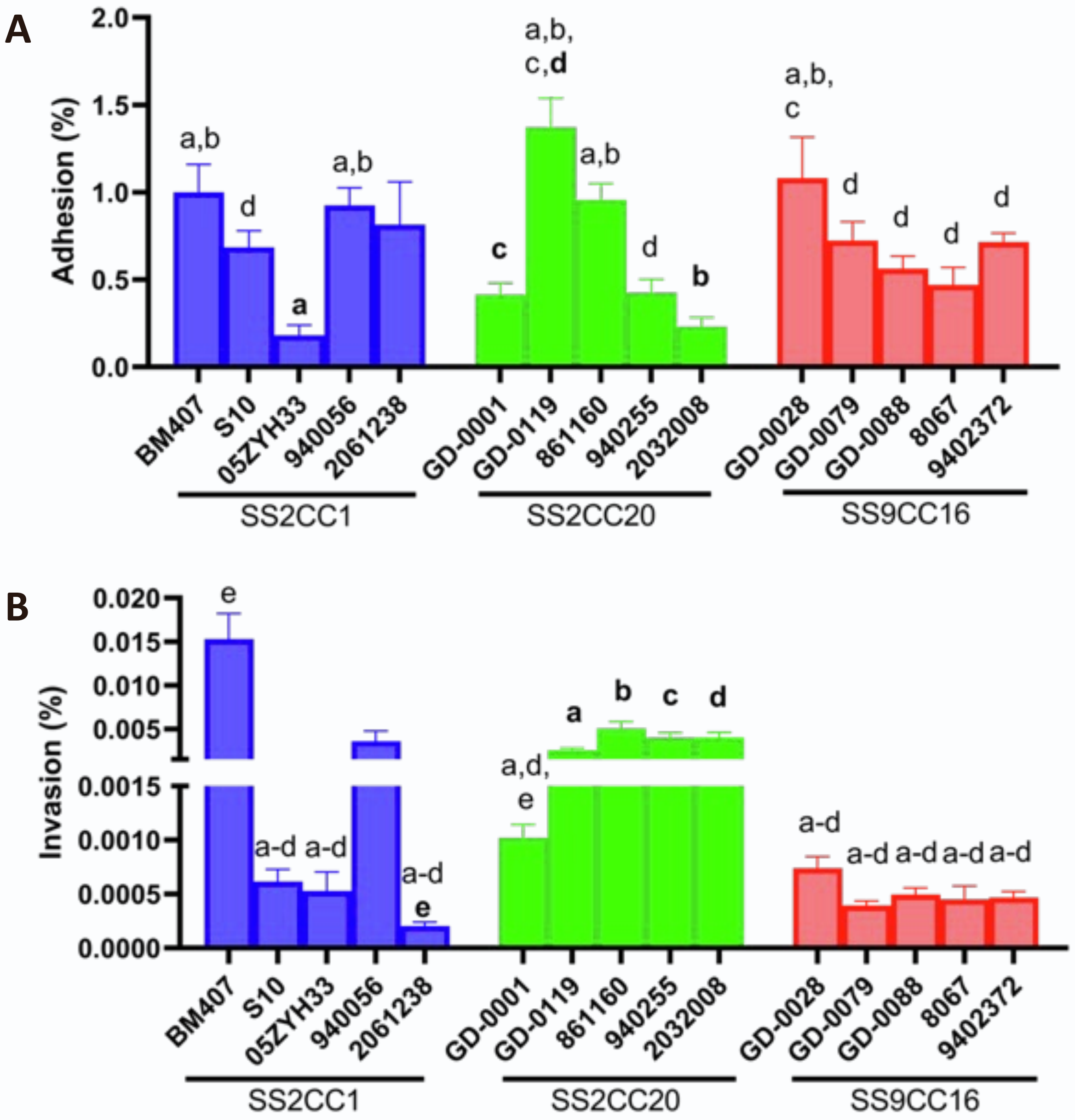

FIG S5 Adhesion to the proximal enteroid monolayer and invasion of proximal epithelial cells of 15 strains from zoonotic (SS2CC1, SS2CC20) and non-zoonotic (SSCC16) genotypes related to Figure 4. (A) Adhesion to proximal enteroid monolayer by individual strains, mean and SEM was plotted. Statistical difference was assessed using a One-way ANOVA with Tukey's multiple comparisons test. Letters denote significant differences to bold letter labelled bar with at least $p<0.05$. (B) Invasion of proximal enteroid monolayer by individual strains, mean and SEM was plotted. Statistical difference was assessed using a Welch's ANOVA with Dunnett's T3 multiple comparisons test. Letters denote significant differences to bold letter labelled bar with at least $p<0.05$.

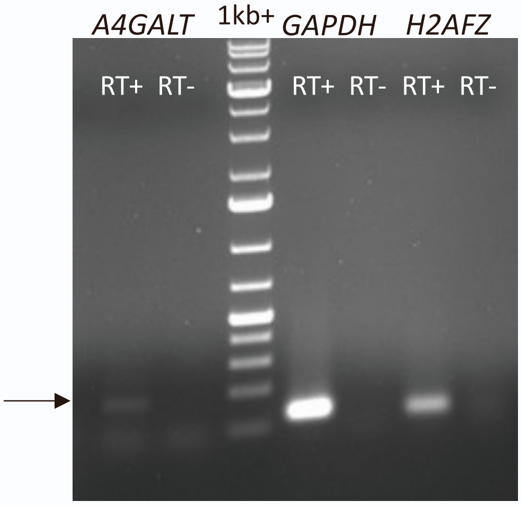

FIG S6 The Gb3 producing enzyme A4GALT is expressed in the proximal enteroid monolayer related to Figure 5. Isolated RNA was reverse transcribed (RT+) or not (RT-), and genomic DNA was removed. Expression of *A4GALT* and previously identified reference genes *GAPDH* and *H2AFZ* [1] was assessed by PCR using transcript specific primers. Amplicons were run on a 1% agarose gel together with the 1kb+ DNA ladder (Invitrogen). Arrow indicates band detected for *A4GALT* in only the RT+ lane.

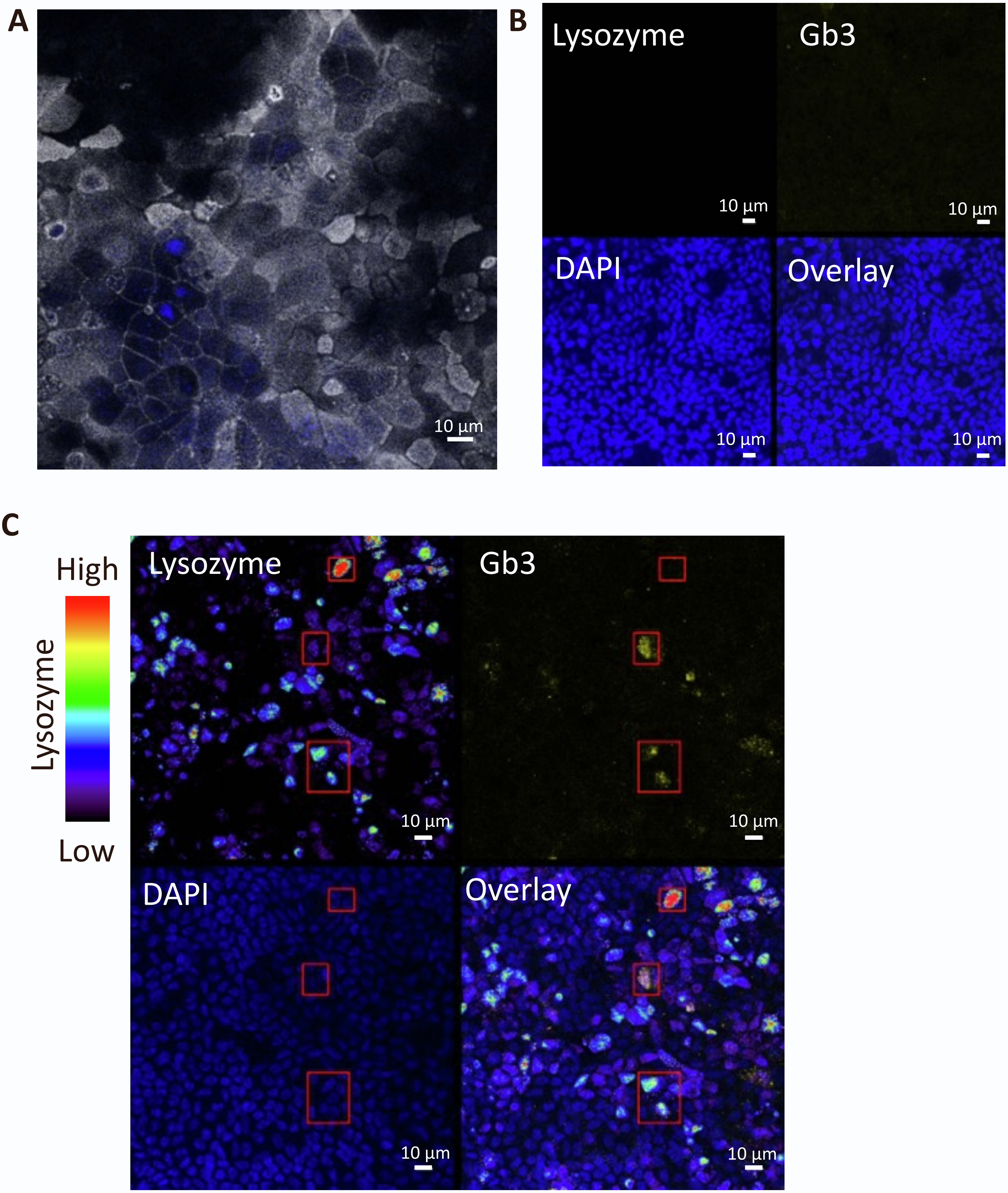

FIG S7 Controls for Gb3-positive cell identification within the proximal enteroid monolayer and lysozyme intensity plot related to figure 5. (A) Proximal enteroid monolayer stained for nuclei (blue) and actin (white), without the primary antibody for Gb3 (yellow) that was visualized by confocal microscopy. (B) Proximal enteroid monolayer stained for nuclei (blue), without primary antibodies against Gb3 (yellow) and Lysozyme (red) that was visualized with confocal microscopy. Micrographs are presented as as maximum projections of a z-stack. (C) Same data as Fig. 5C, but showing Lysozyme staining intensity using pseudocolours.

A

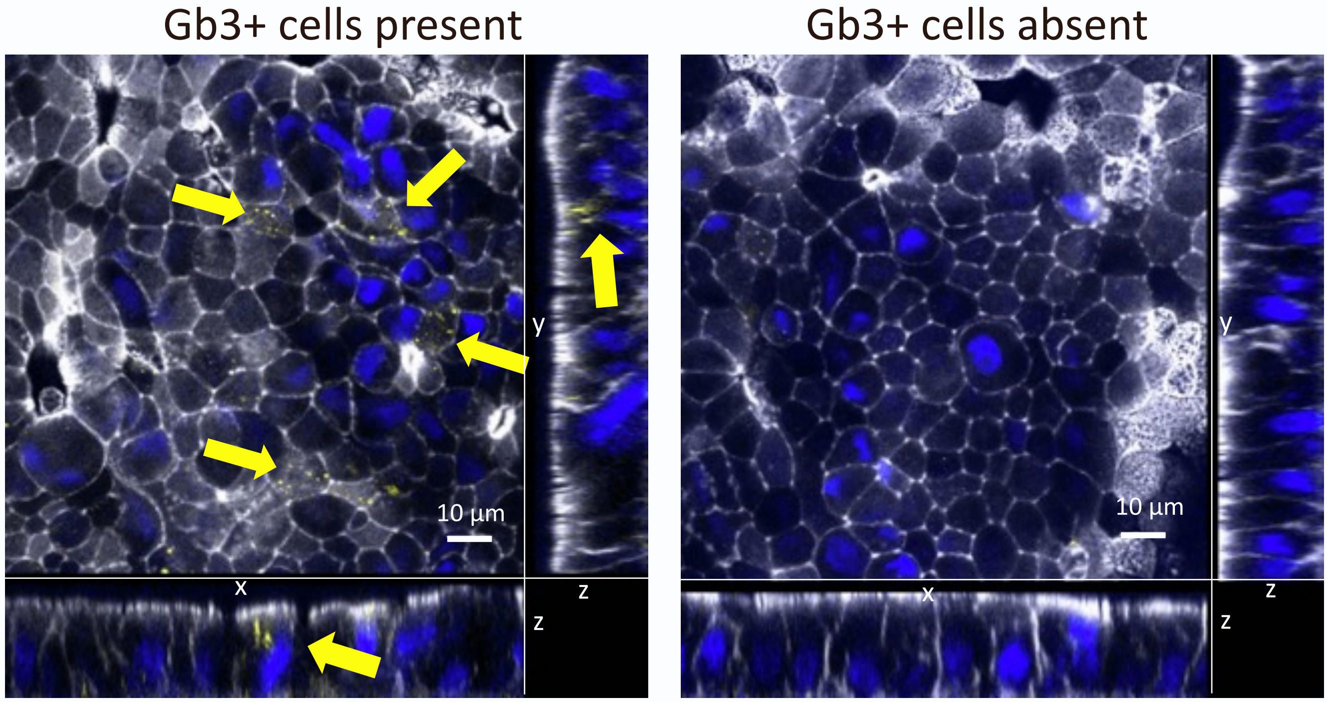

B

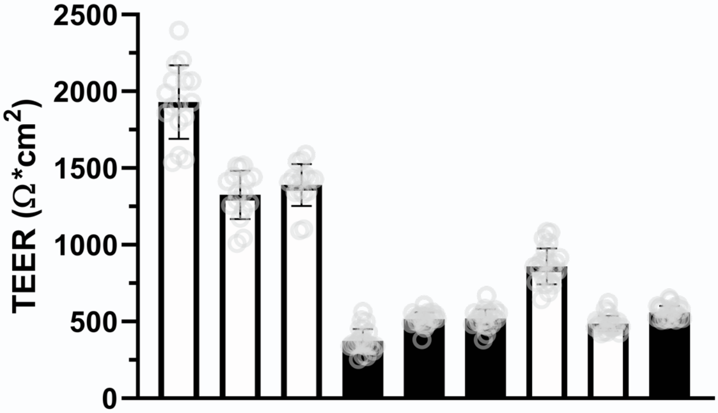

C

Gb3+ cells	-	-	+	+	+	+	-	-	-
PN	9	10	11	12	13	14	18	19	20

FIG S8 Presence of Gb3-positive cells in proximal enteroid monolayer is not associated with TEER or passage number of organoid culture related to figure 5. (A) Proximal enteroid monolayer stained for nuclei (blue), actin (white) and Gb3 (yellow) and visualized with confocal microscopy. Micrographs are presented as z-stack, Gb3-positive cells are indicated by yellow arrow. (B) TEER of proximal enteroid monolayers prior to infection with *S. suis*. Bars denote mean of an independent culture, open bars are monolayers without Gb3-positive cells, filled bars monolayers with Gb3-positive cells. Each symbol is an individual monolayer. (C) Table with passage number (PN) of enteroids used for monolayer seeding, presence of Gb3-positive cells was determined after (mock) infection of proximal enteroid monolayer by FACS.

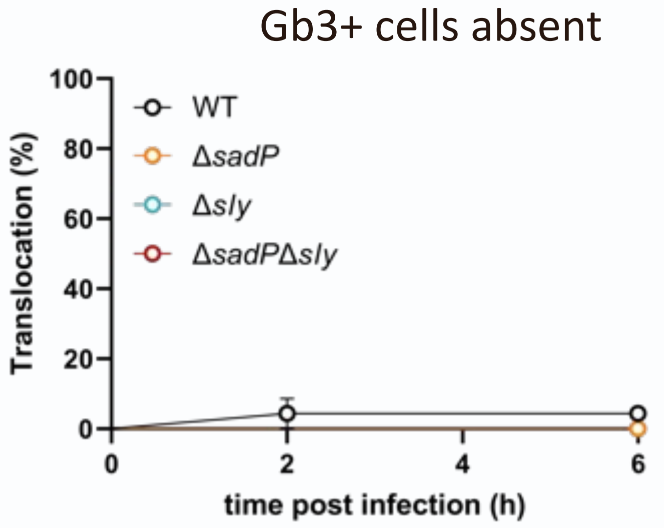

FIG S9 Zoonotic SS2CC1 strain 05ZYH33 translocation across proximal enteroid monolayer does not depend on *sadP* and/or *sly* related to Figure 6. (A) Gb3-positive cells lacking monolayers were apically infected (MOI50) with 05ZYH33 WT*, ΔsadP*, Δ*sly* and Δ*sadP*Δ*sly* and translocation events were recorded every 2 h by plating the basolateral medium and the frequency of translocation, expressed as the number of monolayers in which translocation occurred at 2 h, 4 h and 6 h post infection, relative to the total number of monolayers infected, was plotted. The percentage of monolayers in which translocation occurred was plotted, data was obtained from 10 monolayers per strain. Error bars denote SE. Same WT data was used as in Fig. 5E.

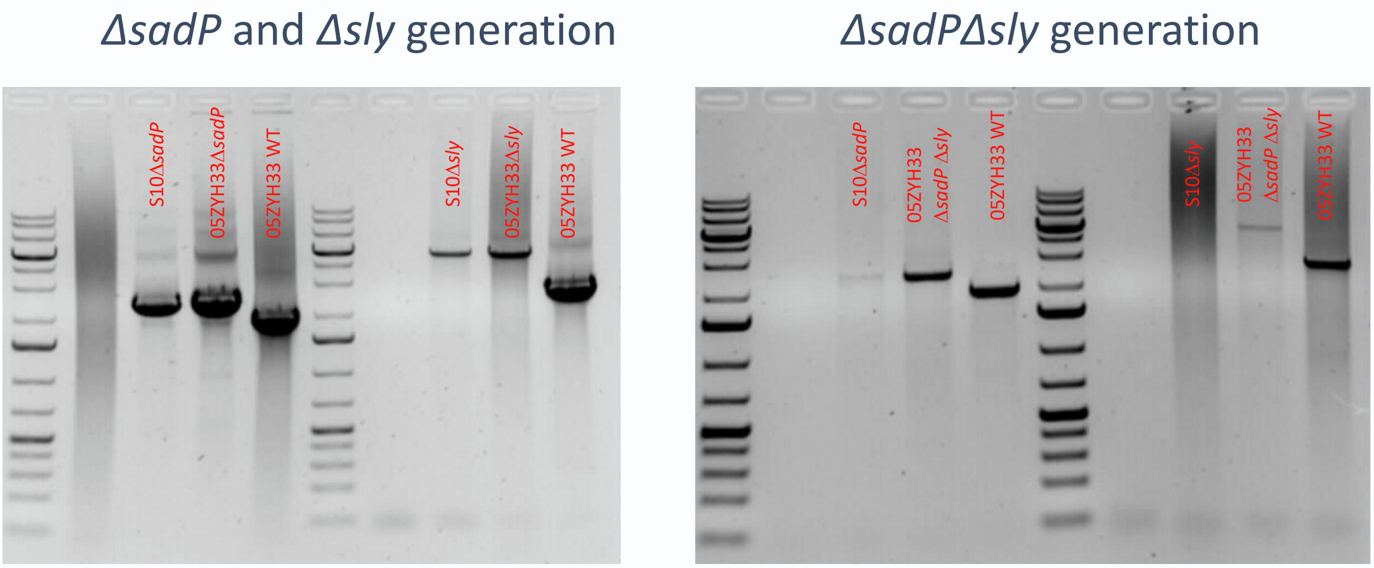

FIG S10 PCR confirmation of *ΔsadP, Δsly* and *ΔsadPΔsly* mutant generation related to STAR methods (Bacterial strains and mutants construction). Amplification of *sadP* and *sly* genomic region with primers listed in table S3. Genomic DNA from 05ZYH33, S10ΔsadP and S10 Δsly was used as controls. Mutants were generated by replace intact *sadP* or *sly* by an interrupted and dysfunctional *sadP* or *sly* genes as previously characterized [2,3]. The increased size of the amplicon compared to the WT and similar size of the amplicon compared to S10Δ*sadP* and S10Δ*sly* confirmed the successful generation of the mutants.

Table S1 Differences in virulence factor presence and absence per strain based on data from Willemse et al. (2016) related to Figure 3.

Strain	Serotype	Clonal Complex	Host	Reference	Hyalluronate Lysase	Serum Opacity factor	SsspA	VirB1-98k	Trag	MRP	NeuB	Permease multidrug transporter	srtF pilus	Extracellular protein factor	RGG-like	CpsF	NisK/R	SalK/R	Prophage SsuCC20p
BM407	2	CC1	Human	4	Absent	Absent	Absent	Present	Present	Absent	Present	Present	Present	Absent	Present	Present	Present	Absent	Absent
S10	2	CC1	Pig	5	Present	Present	Present	Absent	Absent	Present	Present	Present	Present	Present	Present	Present	Absent	Absent	Absent
05ZYH33	2	CC1	Human	6	Present	Present	Present	Present	Present	Present	Present	Present	Present	Present	Present	Present	Present	Present	Absent
940056	2	CC1	Human	7	Present	Present	Present	Present	Present	Present	Present	Present	Present	Present	Present	Present	Absent	Absent	Absent
2061238	2	CC1	Human	7	Present	Present	Present	Present	Present	Present	Present	Present	Present	Present	Present	Present	Present	Absent	Absent
GD0028	9	CC16	Pig	7	Present	Present	Present	Present	Present	Present	Absent	Absent	Absent	Absent	Absent	Absent	Absent	Absent	Absent
GD0079	9	CC16	Pig	7	Present	Present	Present	Present	Absent	Present	Absent	Absent	Absent	Absent	Absent	Absent	Absent	Absent	Absent
GD0088	9	CC16	Pig	7	Present	Present	Present	Present	Present	Present	Absent	Absent	Absent	Absent	Absent	Absent	Absent	Absent	Absent
8067	9	CC16	Pig	7	Present	Present	Present	Present	Present	Present	Absent	Absent	Absent	Absent	Absent	Absent	Absent	Absent	Absent
9402372	9	CC16	Pig	7	Present	Present	Present	Present	Present	Present	Absent	Absent	Absent	Absent	Absent	Absent	Absent	Absent	Absent
GD0001	2	CC20	Pig	7	Present	Present	Present	Present	Present	Absent	Present	Present	Absent	Present	Absent	Present	Present	Absent	Absent
GD0119	2	CC20	Pig	7	Present	Present	Present	Present	Absent	Absent	Present	Present	Absent	Present	Absent	Present	Absent	Present	Present
861160	2	CC20	Human	7	Present	Present	Present	Present	Present	Absent	Present	Present	Absent	Present	Absent	Present	Absent	Present	Present
940255	2	CC20	Human	7	Present	Present	Present	Present	Present	Absent	Present	Absent	Absent	Present	Absent	Present	Present	Absent	Present
2032008	2	CC20	Human	7	Present	Present	Present	Present	Present	Absent	Present	Present	Absent	Present	Absent	Present	Absent	Present	Present

Table S2 List of strains and mutants used in this study related to STAR methods.

Strain	Serotype	Clonal Compl	Host	Reference
BM407	2	CC1	Human	4
S10	2	CC1	Pig	5
05ZYH33	2	CC1	Human	6
940056	2	CC1	Human	7
2061238	2	CC1	Human	7
GD0028	9	CC16	Pig	7
GD0079	9	CC16	Pig	7
GD0088	9	CC16	Pig	7
8067	9	CC16	Pig	7
9402372	9	CC16	Pig	7
GD0001	2	CC20	Pig	7
GD0119	2	CC20	Pig	7
861160	2	CC20	Human	7
940255	2	CC20	Human	7
2032008	2	CC20	Human	7
05ZYH33 Δ*sadP*	2	CC1	Human	this study
05ZYH33 Δ*sly*	2	CC1	Human	this study
05ZYH33 Δ*sadP* Δ*sly*	2	CC1	Human	this study

Table S3 List of primers used to generate sadP and sly mutator fragments related to STAR methods.

Name	Primer sequence '5-'3
sadP-F	GCAAGCAGAAAGTTGTGTCCAG
sadP-R	CTTCATACCCTCTGTCCGTG
sly-F	ACATCATAAAGCCGATTGGA
sly-R	AGGATAGTCTCGCTTGATAATC

Table S4 List of anitbodies and probes used for IF staining related to STAR methods.

Antibody	Supplier	Cat. Nr.	Permeabilization	Dilution
Monoclonal Rat IgG Anti-eCadherin	eBioscience	14-3249-82	Methanol/TritonX	1:100
Monoclonal Rat IgG Anti-ZO-1	Santa Cruz Biotech	R40.76	Methanol/TritonX	1:50
Monoclonal Mouse IgG anti-SOX9	eBioscience	14-9765-82	TritonX	1:200
Polyclonal Rabbit IgG anti-Lysozyme	Invitrogen	PA5-16668	TritonX	1:200
Rabbit serum anti- *S. suis* serotype 2	Statens Serum Institut	22282	Methanol/TritonX	1:1000
Purified mouse IgM anti-human CD77	Biolegend	357102	Methanol/TritonX	1:200
Purified mouse IgM anti-human CD77, FITC-labelled	Biolegend	357103	NA; Flow Cytometry	1:25
Goat anti-rat IgG (H+L)Alexa Fluor 488	Invitrogen	A-11006	Methanol/TritonX	1:500
Phalloidin CruzFluor™ 488 Conjugate	Santa Cruz Biotech	sc-363791	TritonX	1:1000
Goat anti-Rabbit IgG (H+L) Cross-Adsorbed Secondary Antibody, Alexa Fluor 555	Invitrogen	A-21428	Methanol/TritonX	1:500
Goat anti-Mouse IgM (Heavy chain) Cross-Adsorbed Secondary Antibody, Alexa Fluor 555	Invitrogen	A-21426	Methanol/TritonX	1:500
Goat anti-rabbit IgG (H+L)AlexaFluor 633	Invitrogen	A-21070	Methanol/TritonX	1:500
Goat anti-Rat IgG (H+L) Cross-Adsorbed Secondary Antibody, Alexa Fluor 647	Invitrogen	A-21247	Methanol/TritonX	1:500
DAPI	Invitrogen	D1306	Methanol/TritonX	300 nM

Table S5 List of primers used to detect *A4GALT* expression related to STAR methods.

Gene	Forward/Reverse	seq '5-'3
GAPDH	F	AAGGTGAAGGTCGGAGTCAA
	R	AATGAAGGGGTCATTGATGG
H2AFZ	F	CCTCACCGCAGAGGTACTTG
	R	GTTGCAAGTGACGAGGGGTA
A4GALT	F	CACCTCTCTGCAATGGGCTGC
	R	CGTGAACTTGAAGCCGATGATG

References

1. Roodsant, T., Navis, M., Aknouch, I., Renes, I.B., van Elburg, R.M., Pajkrt, D., Wolthers, K.C., Schultsz, C., van der Ark, K.C.H., Sridhar, A., et al. (2020). A Human 2D Primary Organoid-Derived Epithelial Monolayer Model to Study Host-Pathogen Interaction in the Small Intestine. Front. Cell. Infect. Microbiol. *10*, 272. 10.3389/fcimb.2020.00272.
2. Ferrando, M.L., Willemse, N., Zaccaria, E., Pannekoek, Y., Van Der Ende, A., and Schultsz, C. (2017). Streptococcal Adhesin P (SadP) contributes to Streptococcus suis adhesion to the human intestinal epithelium. PLoS One *12*, e0175639. 10.1371/journal.pone.0175639.
3. Benga, L., Fulde, M., Neis, C., Goethe, R., and Valentin-Weigand, P. (2008). Polysaccharide capsule and suilysin contribute to extracellular survival of Streptococcus suis co-cultivated with primary porcine phagocytes. Vet. Microbiol. *132*, 211–219. 10.1016/j.vetmic.2008.05.005.
4. Holden, M.T.G., Hauser, H., Sanders, M., Ngo, T.H., Cherevach, I., Cronin, A., Goodhead, I., Mungall, K., Quail, M.A., Price, C., et al. (2009). Rapid evolution of virulence and drug resistance in the emerging zoonotic pathogen Streptococcus suis. PLoS One *4*, e6072. 10.1371/journal.pone.0006072.
5. Smith, H.E., Damman, M., Van Der Velde, J., Wagenaar, F., Wisselink, H.J., Stockhofe-Zurwieden, N., and Smits, M.A. (1999). Identification and characterization of the cps locus of Streptococcus suis serotype 2: The capsule protects against phagocytosis and is an important virulence factor. Infect. Immun. *67*, 1750–1756. 10.1128/.67.4.1750-1756.1999.
6. Ye, C., Zhu, X., Jing, H., Du, H., Segura, M., Zheng, H., Kan, B., Wang, L., Bai, X., Zhou, Y., et al. (2006). Streptococcus suis sequence type 7 outbreak, Sichuan, China. Emerg. Infect. Dis. *12*, 1203–1208. 10.3201/eid1708.060232.
7. Willemse, N., Howell, K.J., Weinert, L.A., Heuvelink, A., Pannekoek, Y., Wagenaar, J.A., Smith, H.E., Van Der Ende, A., and Schultsz, C. (2016). An emerging zoonotic clone in the Netherlands provides clues to virulence and zoonotic potential of Streptococcus suis. Sci. Rep. *6*, 28984. 10.1038/srep28984.
